# Author Correction: An optochemical tool for light-induced dissociation of adherens junctions to control mechanical coupling between cells

**DOI:** 10.1038/s41467-020-15275-z

**Published:** 2020-03-31

**Authors:** Dirk Ollech, Tim Pflästerer, Adam Shellard, Chiara Zambarda, Joachim Pius Spatz, Philippe Marcq, Roberto Mayor, Richard Wombacher, Elisabetta Ada Cavalcanti-Adam

**Affiliations:** 1 Department of Cellular Biophysics, Max Planck Institute for Medical Research, Jahnstraße 29, D-69120 Heidelberg, Germany; 2 Department of Biophysical Chemistry, Institute of Physical Chemistry, Heidelberg University, INF 253, D-69120 Heidelberg, Germany; 3 Department of Pharmaceutical Chemistry, Institute of Pharmacy and Molecular Biotechnology, Heidelberg University, INF 364, D-69120 Heidelberg, Germany; 4 Department of Cell and Developmental Biology, University College London, London, WC1E 6BT UK; 5 PMMH, CNRS, ESPCI Paris, PSL University, Sorbonne Université, Université de Paris, F-75005 Paris, France; 6 Present Address: Applied Physics Department, Science for Life Laboratory and KTH Royal Technical University, Tomtebodavägen 23A, S-17165 Stockholm, Sweden

**Keywords:** Biophysics, Biotechnology, Chemical biology

Correction to: *Nature Communications* 10.1038/s41467-020-14390-1, published online 24 January 2020

The original version of this Article contained an error in Fig. 7, in which panels s, t and u were described as being treated with Ha-pl-BG. They were treated with Ha-TMP. This has been corrected in both the PDF and HTML versions of the Article.

