# Supplementary material for: An optochemical tool for light-induced dissociation of adherens junctions to control mechanical coupling between cells
Source: Nat Commun. 2020 Jan 24;11:472. doi: 10.1038/s41467-020-14390-1 (PMC6981158; doi:10.1038/s41467-020-14390-1)
Supplement: Supplementary file 1 — Supplementary Information [file 41467_2020_14390_MOESM1_ESM.pdf]

Supplementary Information

An optochemical tool for light-induced dissociation of adherens junctions to control mechanical coupling between cells

Ollech D et al.

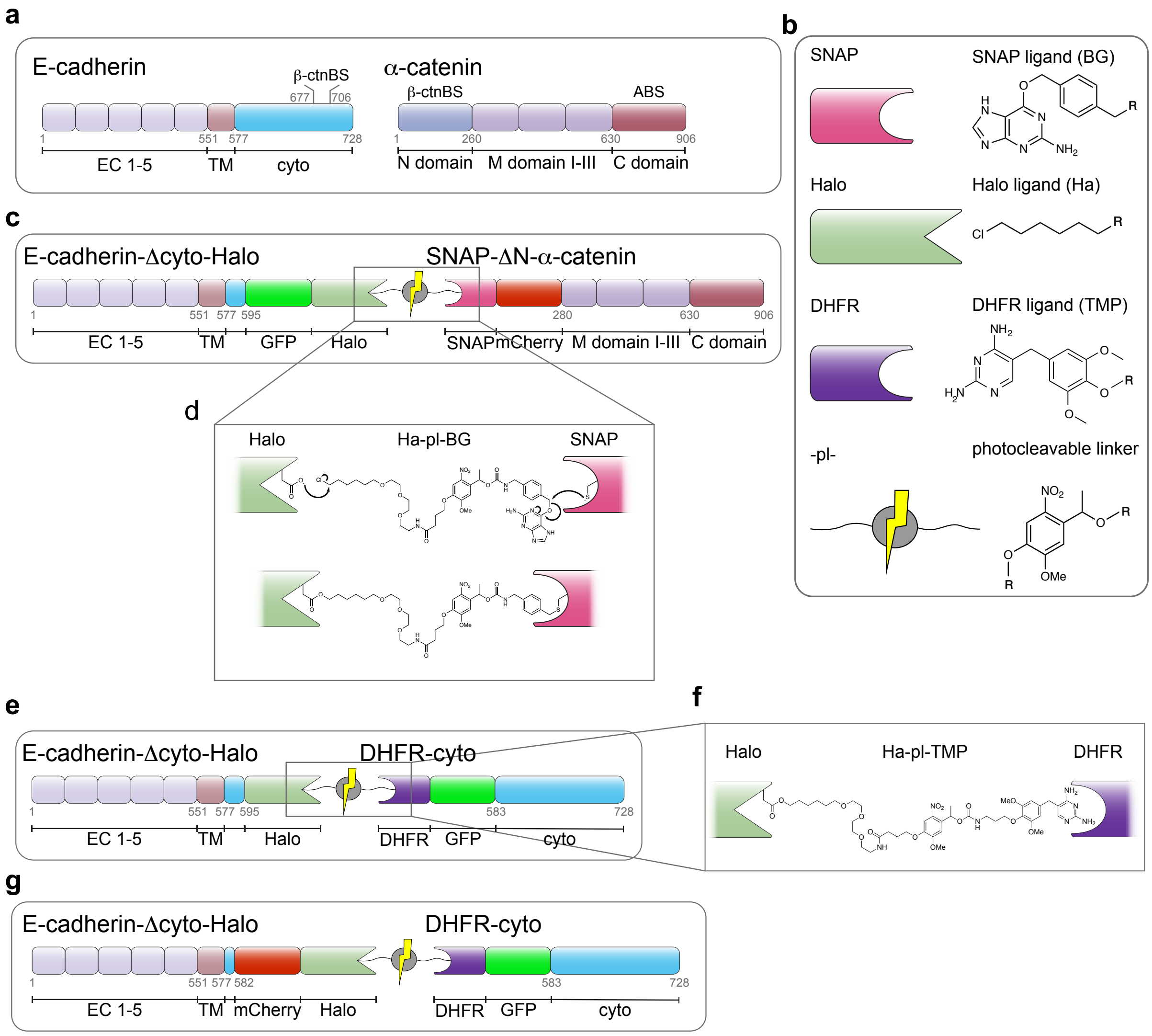

Supplementary Figure 1: Reconstituting the link between E-cadherin and α-catenin with chemical dimerizers. (a) E-cadherin is a membrane spanning adhesion protein with an extracellular part consisting of five homologous domains (EC1-5). The transmembrane region (TM) is connected to the cytosolic domain which carries the β-catenin binding site (β-ctnBS). α-catenin has a β-ctnBS in the N terminal domain, which is followed by a tripartite middle domain (M domain I-III) and the F-actin binding site (ABS). The minimal mechanotransducing connection from the contractile actomyosin network to E-cadherin is maintained by the link to α-catenin *via* β-catenin. **(b)** Chemical structures of the functional groups of the hetero-bifunctional small molecules combining the chloro-hexane (Halo) ligand of Halo tag and the benzyle guanine (BG) ligand of SNAP tag or the dihydrofolate reductase (DHFR) ligand trimethoprim (TMP) *via* a flexible polyethylene linker. The photocleavable linker (pl) contains the light sensitive dimethoxy-nitrophenyle group. **(c)** To establish the link between E-cadherin and α-catenin, most of the cytosolic domain of E-cadherin was replaced with a Halo tag leaving only a 22 amino acid linker sequence that has no binding sites for AJ complex proteins. The N domain of α-catenin was replaced by SNAP tag. For live cell imaging GFP was inserted between E-cadherin and Halo tag and mCherry between SNAP tag and α-catenin domain. **(d)** When the photocleavable dimerizer Ha-pl-BG is added to the culture medium of cells coexpressing E-cadherin-Δcyto-Halo and SNAP-ΔN-α-catenin the SNAP and Halo tag bind their respective ligands covalently, thereby forming a stable link between E-cadherin and α-catenin. **(e)** To reconstitute a split version of E-cadherin, the E-cadherin-Δcyto-Halo is

coexpressed with the cytosolic tail of E-cadherin fused to DHFR and GFP. **(f)** The photocleavable dimerizer Ha-pl-TMP establishes a non-covalent bond with the DHFR tag. **(g)** For *in vivo* experiments, mCherry was inserted in the E-cadherin-Δcyto-Halo before the Halo tag.

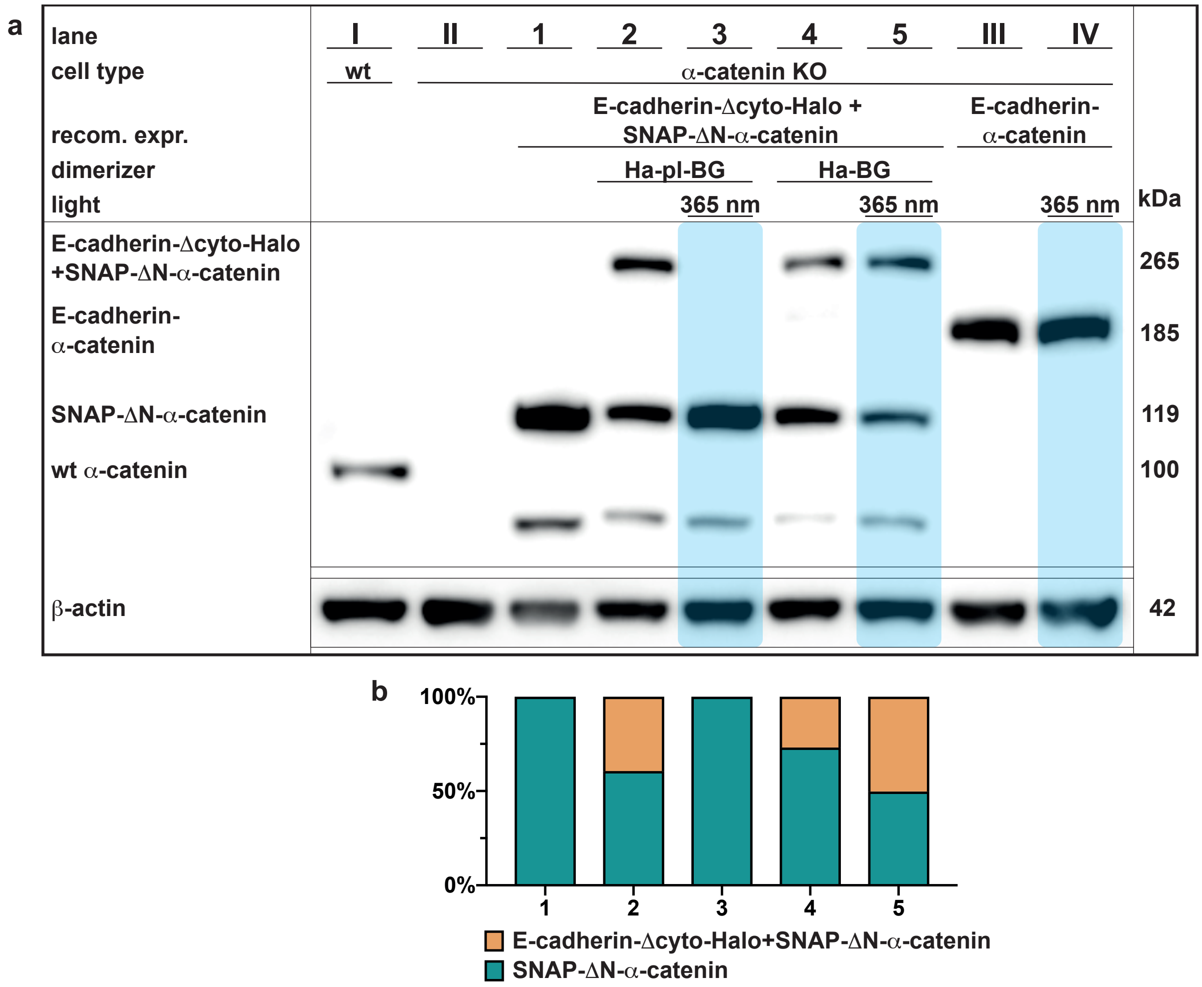

Supplementary Figure 2: Western blot analysis of E-cadherin-α-catenin complexes in α-catenin KO cells and their stability after 365 nm light. (a) Full view of the Western blot presented in Figure 2 c comparing the α-catenin level of A431 wild type cells (lane I) expressing endogenous α-catenin (100 kDa) against untransfected A431 α-catenin KO cells (lane II) expressing no α-catenin and A431 α-catenin KO cells stably coexpressing E-cadherin-Δcyto-Halo (not stained) and SNAP-ΔN-α-catenin (119 kDa, lanes 1-5) or the stable E-cadherin-α-catenin fusion protein (185 kDa, lanes III and IV). Lane 1-5 have been discussed in Figure 2c. The integrity of the E-cadherin-α-catenin fusion protein is not effected by the 365 nm light that was used to cleave Ha-pl-BG. **(b)** Normalized quantification of the unbound SNAP-ΔN-α-catenin (dark green) versus SNAP-ΔN-α-catenin in complex with E-cadherin-Δcyto-Halo (orange). Note that although less then 50% of the SNAP-ΔN-α-catenin proteins are bound, the amount of formed complexes is sufficient to induce morphological changes as presented in Figure 2a,b.

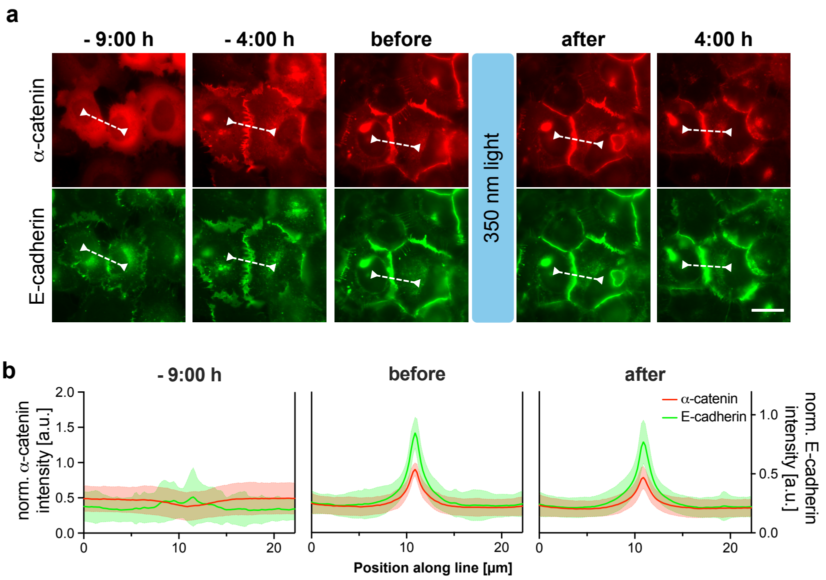

Supplementary Figure 3: Adherens Junctions reconstituted with photostable dimerizer Ha-BG do not dissociate after 350 nm light pulse. (a) Addition of Ha-BG (-9:00 h) recruits the previously cytosolic α-catenin (mCherry-labled, shown in red) to the cell membrane and induces E-cadherin (GFP-labled, shown in green) mediated AJ formation (-4:00 h). Over time AJs change from irregular fringed assemblies into more defined linear structures. As indicated by the unchanged fluorescence intensity profiles, the pulse of 350 nm light has no effect on the E-cadherin-α-catenin complexes (see before and after). Note that the strong rearrangements of AJs (4:00 h) in this time lapse experiment are mainly because of cells undergoing cytokinesis. Interestingly, AJs form as soon the daughter cells start spreading. See also Supplementary Video 2. Scale bar 20 µm. **(b)** Intensity profiles of GFP tagged E-cadherin (green) and mCherry tagged α-catenin (red) are corresponding to the arrow headed dashed lines in the fluorescence images above. The position of the lines has been adjusted for each time point to reflect profiles perpendicular and centered to the cell-cell interface. Mean ± s.d. are shown for *n*=45 cross-sections in multiple fields of view for each time point.

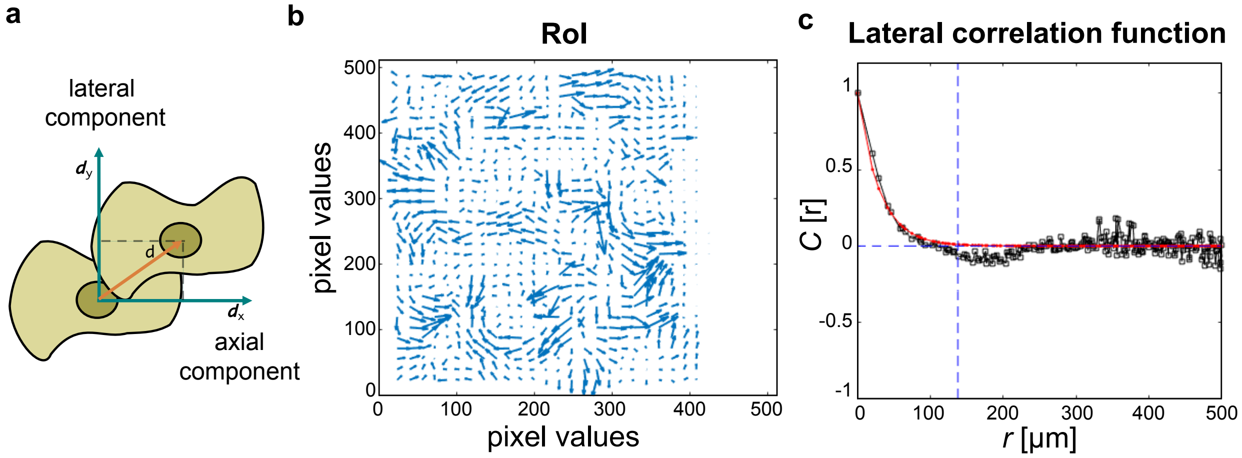

Supplementary Figure 4: PIV analysis. (a) Velocity components evaluated with the PIV analysis: *d*x axial component and *d*y lateral component of the velocity vector *d*. **(b)** Displacement vectors of moving cells between two consecutive phase contrast images are then calculated with the PIV tool. **(c)** We define the correlation length as the distance *r* at first zero-crossing of the correlation function *C*(*r*).

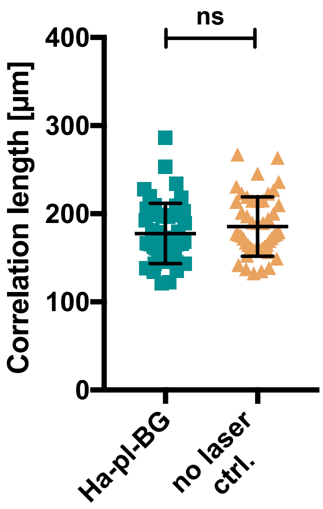

Supplementary Figure 5: Velocity correlation length is stable without photocleavage. Uncleaved control for the migration experiment described in Figure 5 b. PIV analysis of dimerizer treated cells without light-induced dissociation of AJs (no laser ctrl.) shows no significant reduction of collectivity in the observed cells as measured by the velocity correlation length. *n*=48 (12 time points from 4 positions) for cells before (dark green squares) and after (orange triangulars) probed cells were exposed to 405 nm, respectively. Black bars indicate mean ± s.d.; ns: not significant, *p*=0.2634 by unpaired t-test with Welch´s correction.

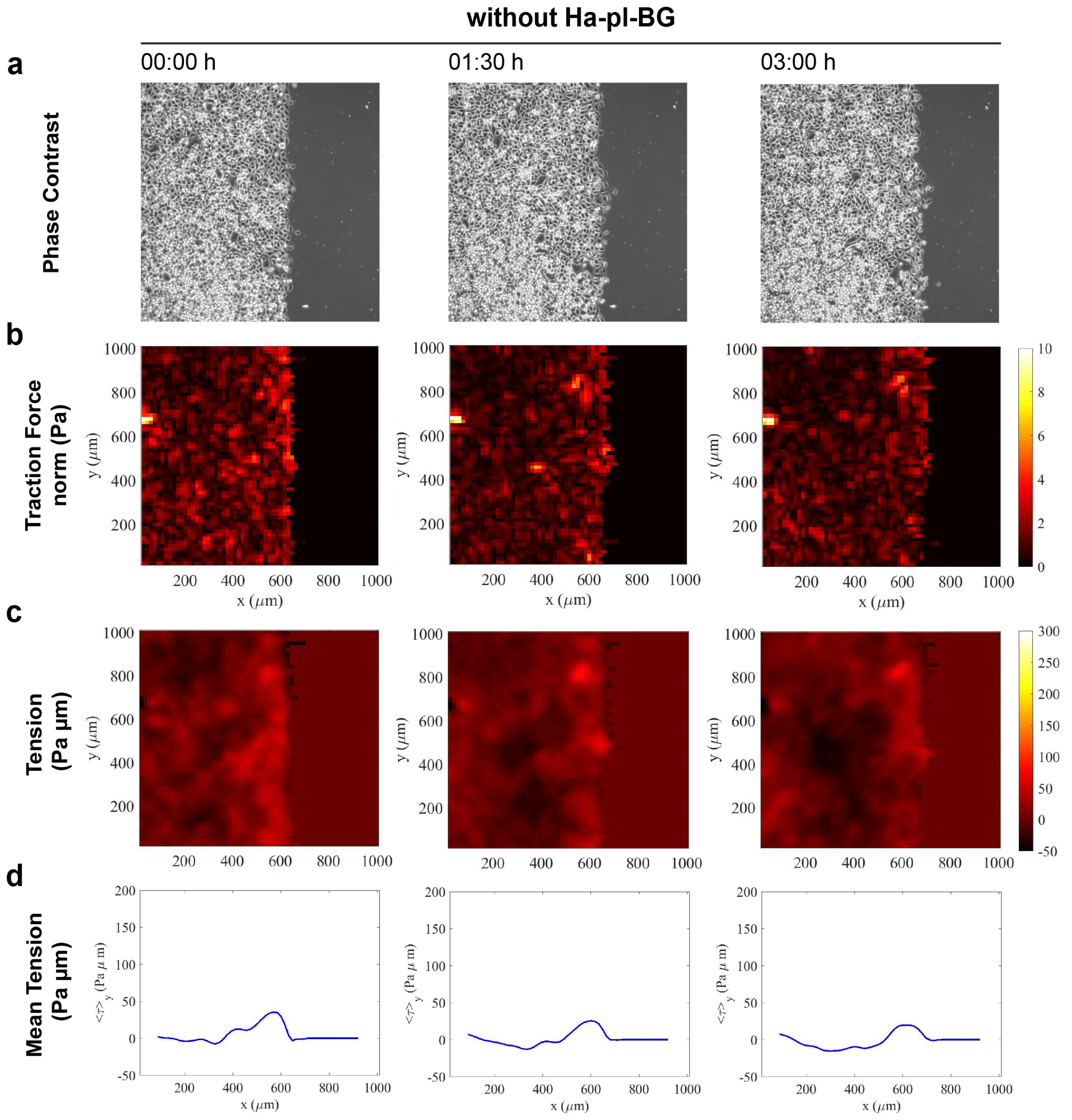

Supplementary Figure 6: In absence of the Ha-pl-BG dimerizer the internal tension of epithelial monolayers decreases over time. (a) Phase contrast images of A431 α-catenin knockout cells. **(b)** Traction Force Microscopy analysis of migrating cells shows that the norm traction forces are maintained as the monolayer migrates. **(c)** Heat maps of the mechanical tension. **(d)** 1D profile of the tension averaged over the direction parallel to the moving front. Internal stresses estimated by BISM on traction force data.

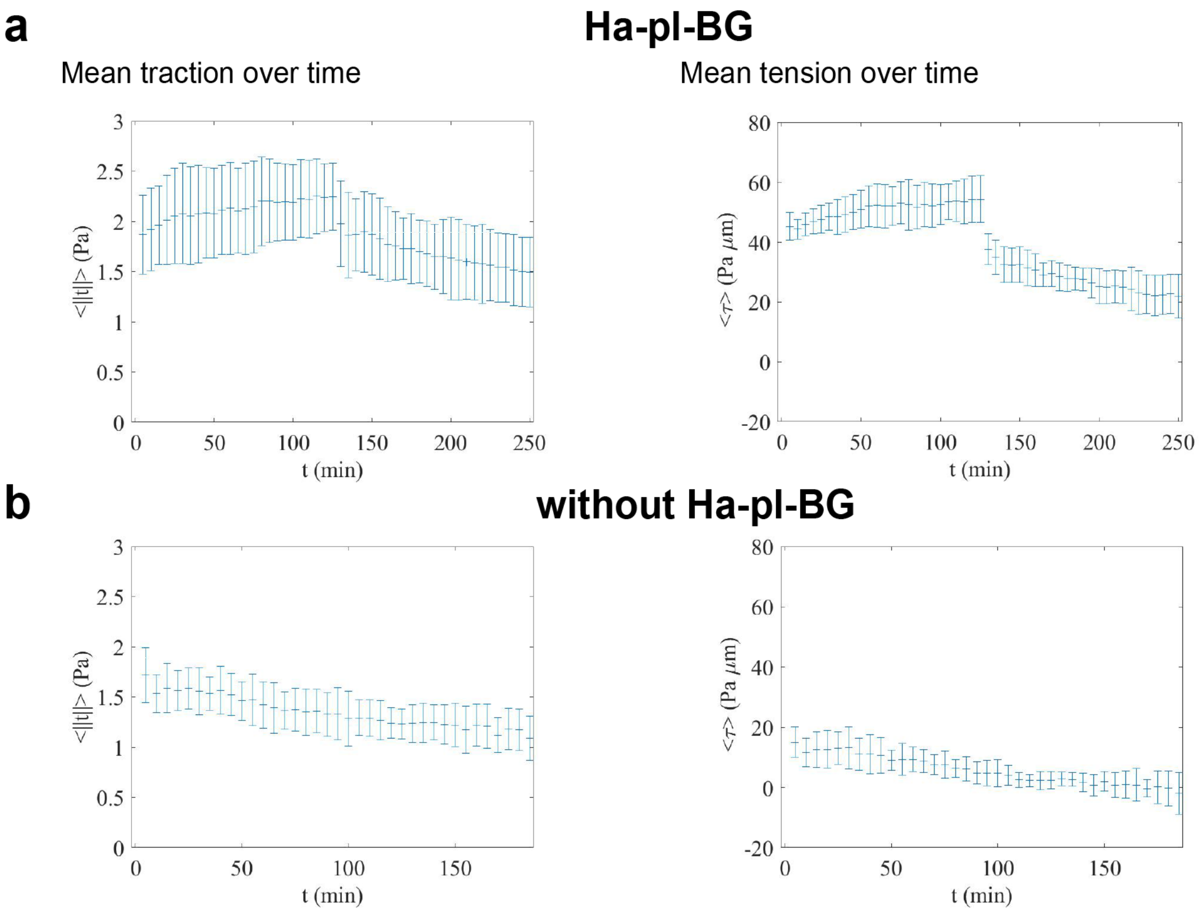

Supplementary Figure 7: Mean traction and tension values computed over time for A431 α-catenin knockout cells migrating **(a)** after addition of the Ha-pl-BG dimerizer and **(b)** in absence of the Ha-pl-BG dimerizer. Error bars indicate s.d. (number of fields analyzed over time *n*=5 in a and *n*=3 in b).

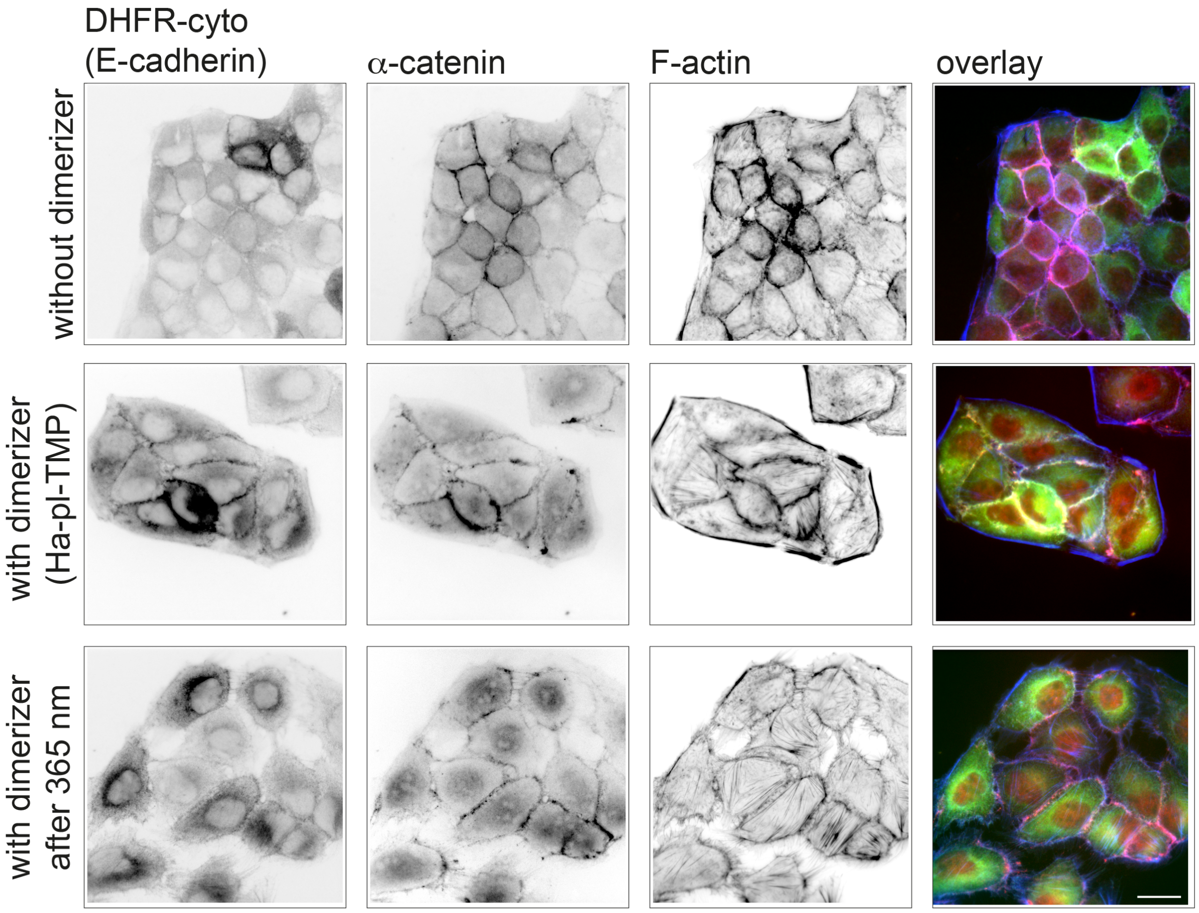

Supplementary Figure 8: Validation of the Ha-pl_TMP dimerizer to reconstitute E-cadherin. MDCK E-cadherin knockout cells were transfected to coexpress E-cadherin-Δcyto-Halo and DHFR-GFP-cyto(E-cadherin). Filamentous actin was labeled using phalloidin conjugated with Alexa647 dye. In absence of the dimerizer, E-cadherin is absent at cell-cell junctions, whereas α-catenin is present at the cell membrane due to association to other cadherins expressed by these cells. Addition of Ha-pl-TMP induces E-cadherin mediated AJ formation which are then disassembled following exposure to 365 nm light for 1 sec. Scale bar 20 μm.

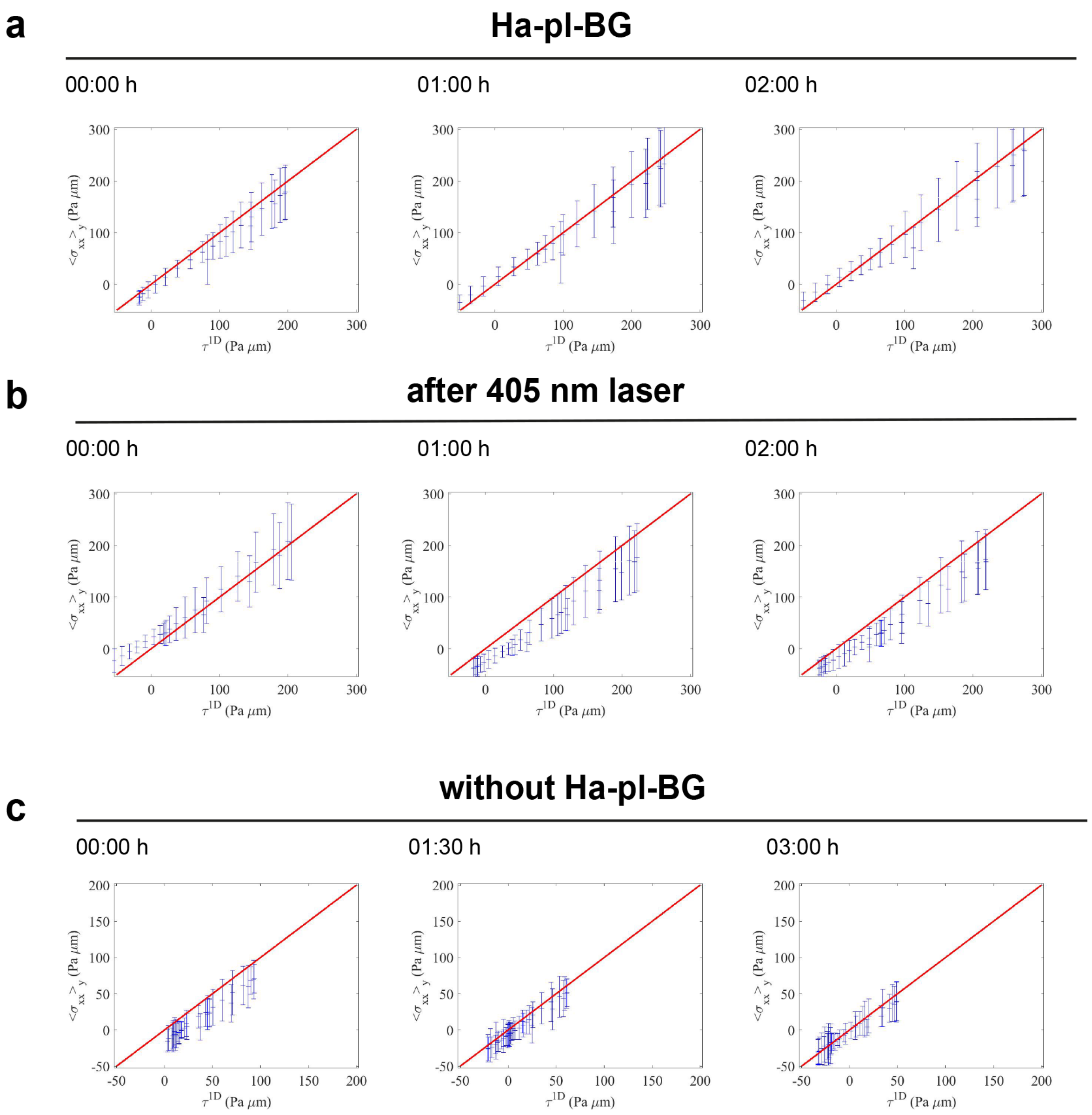

Supplementary Figure 9: Comparison with 1D tension. Plots of $\langle \sigma_{xx}(x,t) \rangle_y$ (y-averaged x-component of the BISM stress) *vs.* 1D tension τ^{1D} (as in Fig. 6 and Supplementary Figure 7). Error bars are standard deviations. The red lines are the bisectors $y = x$ for comparison. Unit: Pa µm.

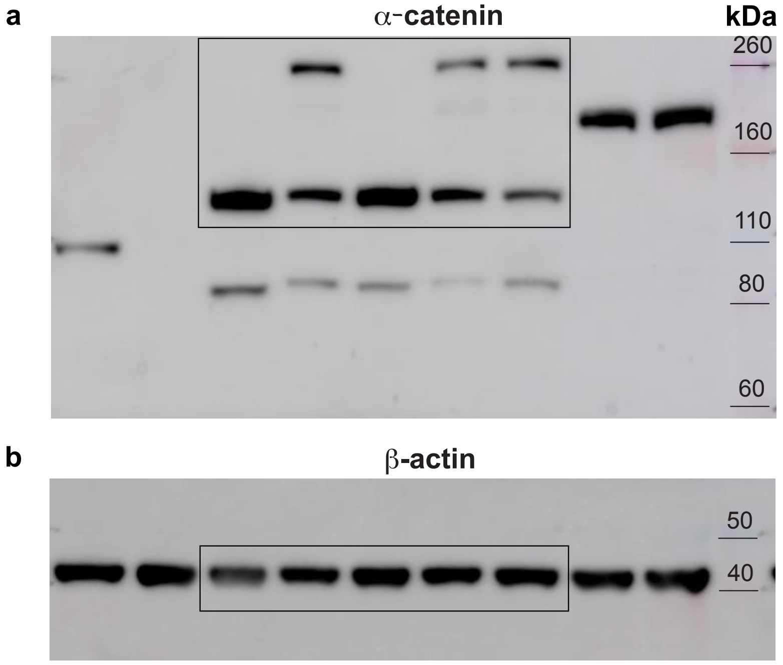

Supplementary Figure 10: Unprocessed Western Blot. Unprocessed images of the Western Blot presented in Figure 2c and Supplementary Figure 2a. The areas presented in Figure 2c are outlined in black. Molecular weight of the colorimetric protein size standard are given on the right in kDa.

Supplementary Methods

Abbreviations

BG	benzylguanine
DCM	dichloromethane
DIPEA	N,N-diisopropylethylamine
DMF	N,N-dimethylformamide
DMSO	dimethyl sulfoxide
eq.	equivalent(s)
ESI	electron spray ionization
EtOAc	ethyl acetate
HPLC	high performance liquid chromatography
HR-MS	high resolution mass spectrometry
NMR	nuclear magnetic resonance spectroscopy
MeCN	acetonitrile
MeOH	methanol
PyBOP	(benzotriazol-1-yloxy)tripyrrolidinophosphonium hexafluorophosphate
RP	reverse phase
RT	room temperature (25 °C)
TEA	trimethylamine
TFA	trifluoroacetic acid
TLC	thin-layer chromatography
TMP	trimethoprim
UV-Vis	Ultraviolet-Visible

1 Chemical Synthesis

1.1 General Information

Chemicals were purchased from Sigma-Aldrich Co. LLC, abcr GmbH & Co. KG, acros Organics and used as received unless otherwise noted. NMR solvents were purchased from euriso-top SAS. Polygram Sil G/UV254 TLC plates from Macherey-Nagel GmbH & Co KG were used for thin layer chromatography. Normal phase column chromatography was performed using silica gel from Fluka with a pore size of 60 Å and a particle size range of 40-63 µm. Solvents were used at p.a. quality.

All compounds were characterized by NMR (^{1}H, ^{13}C) and HR-MS/MS (ESI). Purity was further determined by HPLC-analysis (reinjection of the purified compound) and integration of the UV absorbance signal at λ=256 nm of the chromatogram. HPLC analytics and semi-preparative purifications were done on an Agilent 1100 series HPLC system. Phenomenex Luna 3µ and 5µ C18 reversed-phase columns were used for these purposes (Solvent A: H2O containing 0.1% trifluoroacetic acid (TFA); Solvent B: MeCN containing 0.1% TFA). Collected HPLC fractions were dried by lyophilization. Mass spectrometry was performed on a Bruker microTOF-QII mass spectrometer. NMR spectra were recorded on a Varian Mercury Plus 300 MHz spectrometer or a Varian 500 MHz NMR System. Peak shifts were reported relative to solvent peaks according to Fulmer *et al.*[1]
Purified final compounds were dissolved in DMSO to prepare stock solutions of 200 µM Ha-pl-BG or 40 µM Ha-BG, respectively. The concentration of these solutions was determined from a serial dilution by measuring the absorption at λ= 280 nm using the UV-Vis spectrometer.

1.2 Synthetic schemes

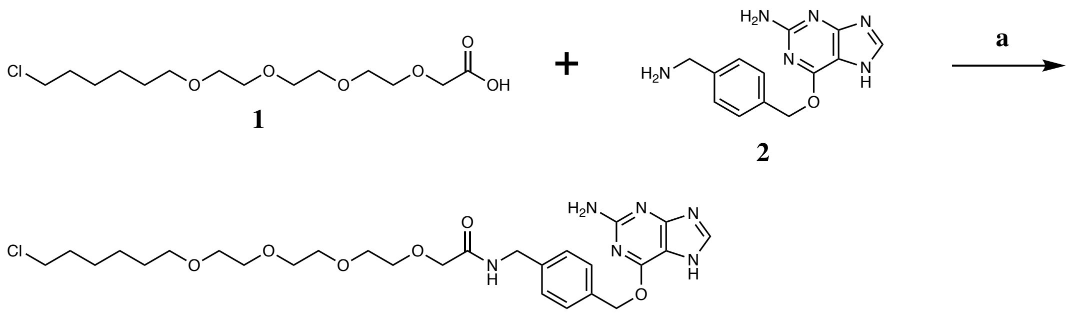

Ha-BG (3)

Synthesis Scheme 1: Ha-BG

Reagents and conditions: (a) i) **1**, PyBOP, DMF, RT, 2 h; ii) **2**, DIPEA, RT, overnight.

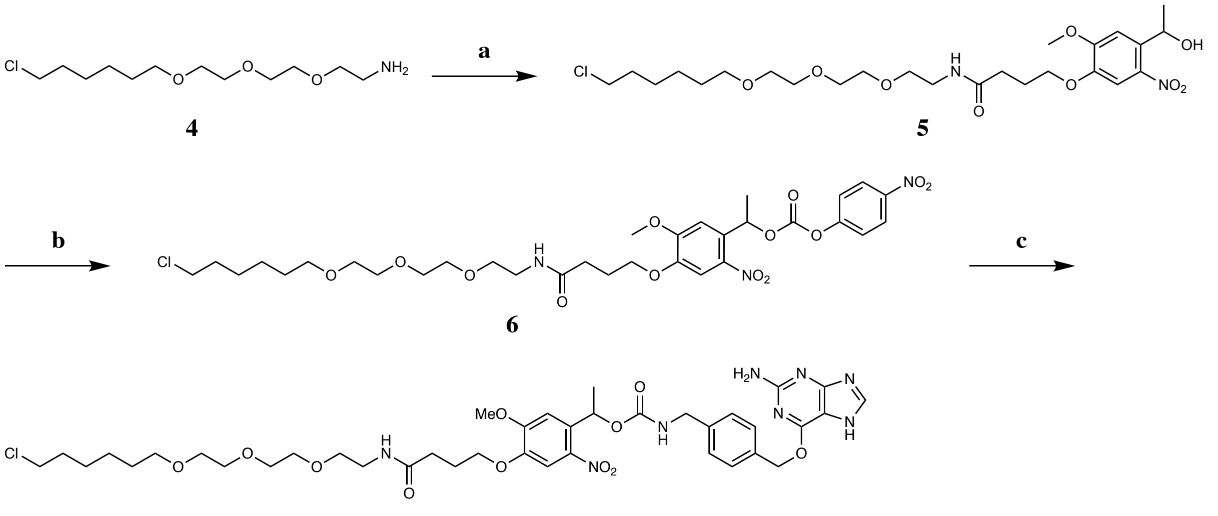

Ha-pl-BG (7)

Synthesis Scheme 2: **Ha-pl-BG**

Reagents and conditions: (a) i) 4-[4-(1-hydroxyethyl)-2-methoxy-5-nitrophenoxy]butyric acid, PyBOP, DMF, RT, 2 h; ii) **4**, DIPEA, RT, overnight; (b) 4-nitrophenyl chloroformate, pyridine, DCM, RT, 2 h; (c) **2**, TEA, DMF, RT, overnight.

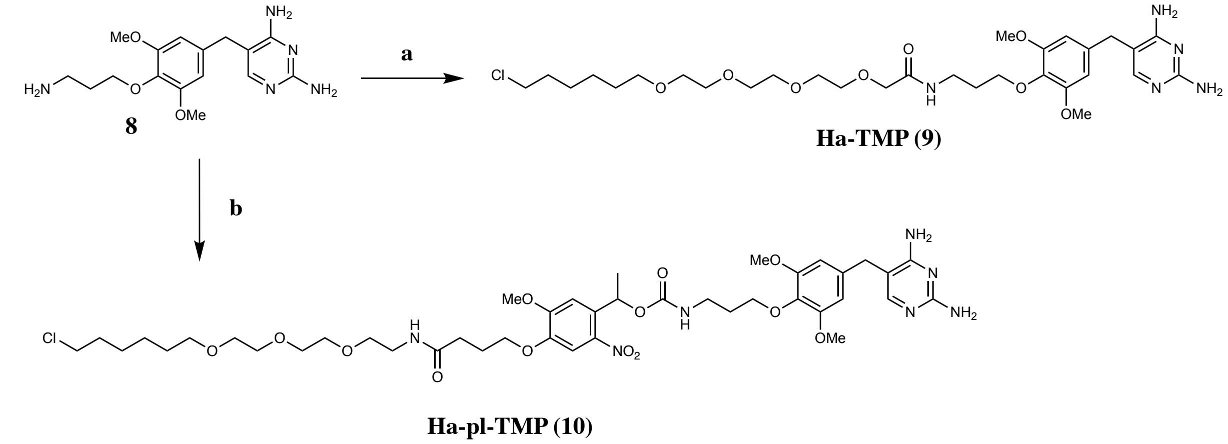

Ha-pl-TMP (10)

Synthesis Scheme 3: **Ha-TMP and Ha-pl-TMP**

Reagents and conditions: (a) i) **1**, PyBOP, DMF, RT, 2 h; ii) **8**, DIPEA, RT, overnight. (b) **6**, TEA, DMF, RT, overnight. **8** was synthesized according to literature.[2]

1.3 Synthesis of compound 1

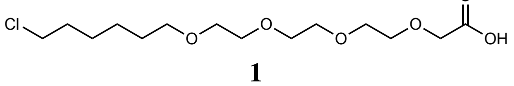

1

Compound **1** was produced according to the synthetic strategy reported previously.[3]

1.4 Synthesis of Ha-BG (3)

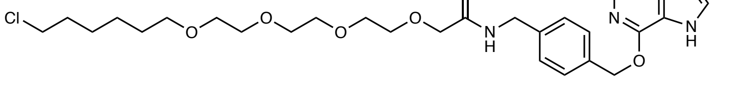

Ha-BG (3)

Compound **1** (78.43 mg, 0.24 mmol, 1.2 eq.) was dissolved in dry DMF (2 ml, 0.1 M) at RT under argon. PyBOP (124.87 mg, 0.24 mmol, 1.2 eq.) was added and the mixture was stirred for 2 h. DIPEA (41.8 µl, 0.24 mmol, 1.2 eq.) and 6-((4-(aminomethyl)benzyl)oxy)-9H-purin-2-amine **5** (54.06 mg, 0.2 mmol, 1.0 eq.) were added and stirring continued over night at RT. The mixture was poured into water (70 ml) and extracted with EtOAc (2x 50 ml). The combined organic fractions were washed with water (20 ml) and dried over $MgSO_4$. All volatile components were removed under reduced pressure. The crude product was purified via flash chromatography (DCM:MeOH 20:1 then 10:1 *v*/*v*) to yield 49.06 mg (42 %) of compound **Ha-BG (3)** as a yellowish oily residue. A fraction was further purified via semi-preparative RP-HPLC to yield a white solid with 98 % purity (retention time= 19.22 min on analytical column). The extinction coefficient of Ha-BG (**3**) at 280 nm was assumed to be identical to $BG_{\varepsilon 280}$: Ha-$BG_{\varepsilon 280}$= 7.1 $mM^{-1}cm^{-1}$.

^{1}H NMR (300 MHz, CD_3OD) δ=7.85 (s, 1H), 7.46 (d, J=8.0, 2H), 7.30 (d, J=8.0, 2H), 5.51 (s, 2H), 4.44 (s, 2H), 4.03 (s, 2H), 3.70-3.59 (m, 4H), 3.58-3.41 (m, 9H), 3.41-3.33 (m, 2H), 1.76-1.62 (m, 2H), 1.50 (m, J=13.7, 6.6, 2H), 1.43-1.23 (m, 4H).

^{13}C NMR (75 MHz, CD_3OD) δ=171.12, 159.88, 159.66, 155.28, 138.21, 135.25, 128.02, 127.03, 127.01, 111.53, 70.46, 70.41, 69.81, 69.76, 69.64, 69.58, 69.43, 69.35, 67.01, 44.08, 41.64, 32.07, 28.86, 26.06, 24.82, 24.78.

HR-MS (ESI pos.) *m/z*: calculated for $C_{27}H_{38}ClN_6O_6$+Na^+ $[M+Na]^+$: 601.2512, measured: 601.2524.

1.5 Synthesis of compound 4

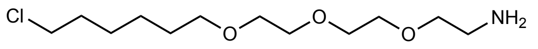

4

Compound **4** was synthesized according to the literature[3] with minor changes. Synthesis was performed as a three step synthesis starting from the 2-(2-(2-azidoethoxy)ethoxy)ethanol, which was obtained after synthesis according to Fernandez-Menagia et al.[4]

1.6 Synthesis of compound 5

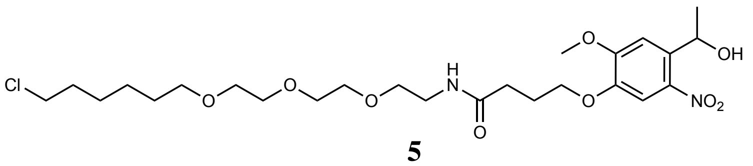

5

4-[4-(1-Hydroxyethyl)-2-methoxy-5-nitrophenoxy]butyric acid (59.86 mg, 0.20 mmol, 1.0 eq.) and PyBOP (124.90 mg, 0.24 mmol, 1.2 eq.) were dissolved in dry DMF (10 ml, 0.02 M) with constant stirring for 2.5 h at RT under argon in the dark. Compound **4** (64.27 mg, 0.24 mmol, 1.2 eq.) was dissolved in DMF (1 ml, 0.24 mM). DIPEA (41.8 µl, 0.24 mmol, 1.2 eq.) and the solution of **4** were added drop wise to the reaction mixture and stirring continued for 20 h under argon at RT in the dark. The crude mixture was poured into water (70 ml) and extracted with EtOAc (3x 50 ml). The combined organic phases were washed with brine (30 ml) and dried over $MgSO_4$ followed by evaporation under reduced pressure. Flash chromatography purification on silica gel (DCM:MeOH 30:1 *v*/*v*) gave 93.49 mg (85 %) of a yellow oily residue.

^{1}H NMR (300 MHz, CD_3OD) δ=7.56 (s, 0H), 7.38 (s, 1H), 5.44 (q, *J*=6.2, 0H), 4.07 (t, *J*=6.2, 1H), 3.95 (s, 2H), 3.62-3.50 (m, 6H), 3.45 (t, *J*=6.5, 1H), 3.36 (t, 1H), 2.41 (t, *J*=7.4, 2H), 2.15-2.05 (m, 1H), 1.79-1.67 (m, 1H), 1.61-1.51 (m, 1H), 1.49-1.32 (m, 4H).

^{13}C NMR (75 MHz, CD_3OD) δ=173.68, 153.80, 146.60, 139.07, 137.36, 108.50, 108.34, 70.51, 69.93, 69.91, 69.61, 69.46, 68.91, 68.06, 64.57, 55.12, 44.09, 38.79, 32.10, 31.80, 28.90, 26.09, 24.85, 24.83, 23.63.

HR-MS (ESI pos.) *m/z*: calculated for $C_{25}H_{42}ClN_2O_9^+$ $[M+H]^+$: 549.2573, measured: 549.2548, calculated for $C_{25}H_{41}ClN_2NaO_9^+$ $[M+Na]^+$: 571.2393, measured: 571.2393, calculated for $C_{25}H_{41}KClN_2O_9^+$ $[M+K]^+$: 587.2132, measured: 587.2123.

1.7 Synthesis of compound 6

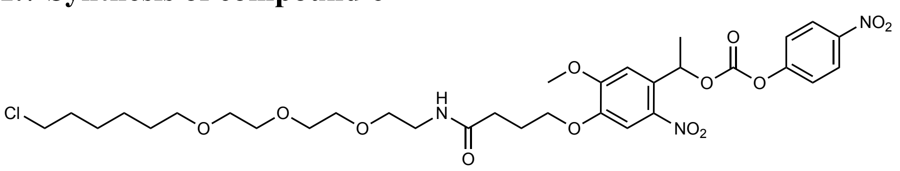

6

A solution of 4-nitrophenyl chloroformate (40.30 mg, 0.20 mmol, 1.2 eq.) in dry DCM (1 ml, 0.2 mM) was added drop wise to a solution of compound **5** (93.49 mg, 0.17 mmol, 1 eq.) and pyridine (34,4 µl, 0.43 mmol, 2.5 eq.) in dry DCM (1 ml) under argon at RT with constant stirring. The mixture turned turbid upon addition of the chloroformate but clarifies immediately. The reaction was let run at RT under argon in the dark for 2 h. All volatile compartments were removed under reduced pressure. Purification by flash chromatography (DCM:MeOH 20:1 *v*/*v*) yielded 112.3 mg (92 %) of the activated alcohol **6** that appeared as a yellow oil.

^{1}H NMR (300 MHz, CD_3OD) δ=8.27-8.19 (m, 1H), 7.58 (s, 1H), 7.43-7.34 (m, 1H), 7.18 (s, 0H), 6.40 (q, *J*=6.4, 1H), 4.07 (t, *J*=6.2, 1H), 3.96 (s, *J*=4.0, 2H), 3.61-3.47 (m, 6H), 3.42 (t, *J*=6.5, 1H), 3.35 (t, *J*=5.4, 1H), 2.41 (t, *J*=7.3, 2H), 2.16-2.04 (m, 2H), 1.76-1.65 (m, 2H), 1.59-1.49 (m, 2H), 1.46-1.29 (m, 4H).

^{13}C NMR (75 MHz, CD_3OD) δ=173.60, 155.24, 153.97, 151.41, 147.58, 145.21, 139.79, 130.69, 124.63, 121.56, 108.41, 107.84, 73.16, 70.49, 69.92, 69.90, 69.59, 69.45, 68.89, 68.08, 55.39, 44.09, 38.79, 32.07, 31.75, 28.88, 26.07, 24.83, 24.73, 20.34.

HR-MS (ESI pos.) *m/z*: calculated for $C_{32}H_{44}ClN_3NaO_{13}^+$ $[M+Na]^+$: 736.2455, measured: 736.2454.

1.8 Synthesis of Ha-pl-BG (7)

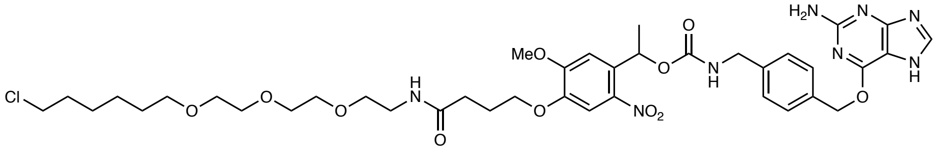

Ha-pl-BG (7)

TEA (18 µl, 130 µmol, 2 eq.) was added to a solution of **6** (46.6 mg, 65 µmol, 1 eq.) in dry DMF (1 ml, 65 µM) at RT under argon with constant stirring. A homogenous suspension of **2** (20.00 mg, 74 µmol, 1.1 eq.) in dry DMF (1 ml) was added drop wise and stirred for 20 h at RT under argon in the dark. After evaporation of volatile compartments the crude mixture was purified *via* flash chromatography on silica gel (DCM:MeOH 25:1 *v*/*v*, then 20:1 *v*/*v*, then 10:1 *v*/*v*). 49 mg (89 %) of a bright yellow oil were obtained. Analysis by RP-HPLC showed 96 % purity (retention time= 24.68 min). The extinction coefficient of Ha-pl-BG (**7**) at 280 nm

was calculated from the individual extinction coefficients of BG and the photolinker unit (pl) moiety: $pl_{\varepsilon 280}$= 4.2 $mM^{-1}cm^{-1}$; $BG_{\varepsilon 280}$= 7.1 $mM^{-1}cm^{-1}$; Ha-pl-$BG_{\varepsilon 280}$= 11.3 $mM^{-1}cm^{-1}$.

^{1}H NMR (500 MHz, CD_3OD) δ = 7.83 (s, 1H), 7.60 (s, 1H), 7.42 (d, J=8.0, 2H), 7.21 (d, J=8.0, 2H), 7.13 (s, 1H), 6.27 (d, J=6.5, 1H), 5.50 (d, J=2.3, 2H), 4.27 (s, 1H), 4.18 (s, 1H), 4.06 (t, J=6.2, 2H), 3.83 (s, 3H), 3.59-3.51 (m, 10H), 3.49 (d, J=6.7, 2H), 3.42 (t, J=6.6, 2H), 3.36 (d, J=5.6, 1H), 2.40 (d, J=7.5, 2H), 2.10 (s, 2H), 1.74-1.66 (m, 2H), 1.58 (d, J=6.5, 3H), 1.55-1.50 (m, 2H), 1.36 (dd, J=29.2, 7.0, 4H).

^{13}C NMR (126 MHz, CD_3OD) δ=173.95, 160.24, 156.53, 154.21, 147.22, 139.57, 139.15, 135.51, 133.99, 128.20, 126.95, 108.81, 107.92, 70.72, 70.13, 70.11, 69.82, 69.67, 69.11, 68.60, 68.31, 67.20, 55.41, 53.39, 48.43, 44.28, 43.70, 39.02, 32.30, 32.06, 29.10, 26.29, 25.05, 20.97.

HR-MS (ESI pos.) *m/z*: calculated for $C_{39}H_{53}ClN_8NaO_{11}^+$ $[M+Na]^+$: 867.3415, measured: 867.3395.

1.9 Synthesis of Ha-TMP (9)

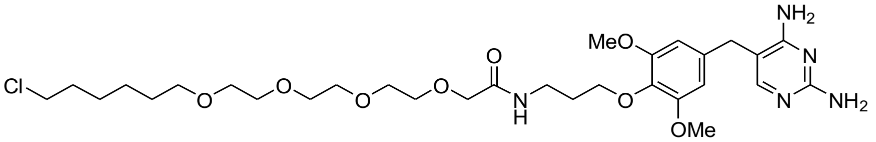

Ha-TMP (9)

4 (78.43 mg, 0.24 mmol, 1.2 eq.) was dissolved in dry DMF (2 ml, 0.1 M) at RT under Ar followed by addition of PyBOP (124.90 mg, 0.24 mmol, 1.2 eq.). The mixture was stirred for 2 h before DIPEA (41.8 µl, 0.24 mmol, 1.2 eq.) and **8** (66.68 mg, 0.2 mmol, 1.0 eq.) were added. **8** was synthesized according to literature.[2] The reaction mixture was stirred over night at RT under Ar for 12 h. The reaction mixture was poured into water (70 ml) and extracted with EtOAc (2x 50 ml). The combined organic fractions were washed with water (20 ml) and dried over $MgSO_4$. After evaporation of solvents the crude mixture was purified *via* flash chromatography on silica gel (DCM:MeOH 20:1 then 10:1 v/v) to yield 36 mg (28 %) of **Ha-TMP (9)** as a clear colourless oil. Further purification via RP-HPLC yielded a white solid of >95 % purity (determined by analytical HPLC). The extinction coefficient of Ha-TMP **(9)** at 280 nm was assumed to be identical to the extinction coefficient of TMP: Ha-$TMP_{\varepsilon 280}$= 3.9 $mM^{-1}cm^{-1}$.

^{1}H NMR (500 MHz, CD_3OD) δ=7.51 (s, 1H), 6.54 (s, 2H), 3.96 (t, 4H), 3.78 (s, *J*=6.9, 1.0, 6H), 3.63 (s, *J*=6.4, 4H), 3.59-3.48 (m, 14H), 3.44 (t, *J*=6.6, 2H), 1.92-1.87 (m, 2H), 1.78-1.68 (m, *J*=10.5, 4.0, 2H), 1.59-1.53 (m, 2H), 1.48-1.40 (m, *J*=14.5, 7.7, 2H), 1.39-1.33 (m, *J*=14.0, 6.7, 2H).

^{13}C NMR (126 MHz, CD_3OD) δ=171.05, 163.13, 161.31, 153.47, 153.41, 135.25, 134.96, 106.84, 105.28, 71.30, 70.75, 70.59, 70.09, 70.01, 69.88, 69.82, 69.71, 63.00, 55.16, 44.30, 36.48, 33.08, 32.33, 29.29, 29.12, 26.31, 25.07.

HR-MS (ESI pos.) *m/z*: calculated for $C_{30}H_{49}ClN_5O_8^+$ $[M+H]^+$: 642.3264, measured: 642.3242, calculated for $C_{30}H_{48}ClN_5NaO_8^+$ $[M+Na]^+$: 664.3084, measured: 664.3076.

1.10 Synthesis of Ha-pl-TMP (10)

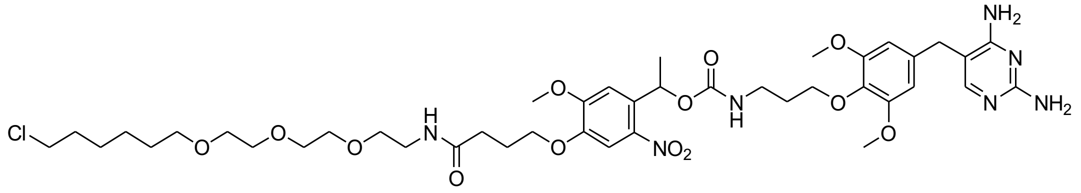

Ha-pl-TMP (10)

To solution of **6** (23.8 mg, 33.3 µmol, 1 eq.) in dry DMF (1 ml, 33.3 µM) triethylamine (18.6 µl, 133.2 µmol, 4 eq.) and **8** (12.22 mg, 36.7 µmol, 1.1 eq.) were added and the reaction mixture was stirred at RT under argon in the dark. After 20 h solvents were evaporated and the resulting crude was purified *via* flash chromatography on silica gel (DCM:MeOH 10:1 *v*/*v*) to yield 13.18 mg (40 %) of **Ha-pl-TMP (10)** as a colourless oil. Analytic RP-HPLC revealed >95 % purity. The extinction coefficient of Ha-pl-TMP **(10)** at 280 nm was calculated from the individual extinction coefficients of TMP and the photolinker (pl) moiety: Halo-pl-$TMP_{\varepsilon 280}$= 8.1 $mM^{-1}cm^{-1}$.

^{1}H NMR (300 MHz, CD_3OD) δ = 7.60 (s, 1H), 7.51 (s, 1H), 7.12 (s, 1H), 6.51 (s, 2H), 6.27 (q, *J*=6.3, 1H), 4.07 (t, *J*=6.2, 2H), 4.01 – 3.88 (m, 2H), 3.83 (s, 3H), 3.76 (s, 6H), 3.63 (s, 2H), 3.61 – 3.49 (m, 13H), 3.44 (t, *J*=6.5, 2H), 3.36 (t, *J*=4.7, 3H), 2.98 (s, 1H), 2.40 (t, *J*=7.4, 2H), 2.16 – 2.04 (m, 2H), 1.86 – 1.76 (m, 2H), 1.71 (dd, *J*=14.5, 6.7, 2H), 1.61 – 1.50 (m, 5H), 1.48 – 1.30 (m, 4H).

^{13}C NMR (75 MHz, CD_3OD) δ=173.68, 153.94, 153.73, 153.03, 146.99, 134.71, 108.53, 106.46, 105.03, 70.76, 70.48, 69.91, 69.88, 69.59, 69.44, 68.87, 68.06, 68.00, 55.11, 54.86, 48.19, 47.90, 47.62, 47.34, 47.05, 46.77, 46.49, 44.05, 38.77, 32.83, 32.07, 31.78, 28.88, 26.06, 24.82, 24.78, 20.81.

HR-MS (ESI pos.) *m/z*: calculated for $C_{42}H_{63}ClN_7O_{13}^+$ $[M+H]^+$: 908.4167, measured: 908.4146.

2 Molecular cloning

ssDNA oligos were ordered from IDT in standard desalting grade. All ligation sites have been sequenced. pERB217 was a gift from David Chenoweth & Michael Lampson (Addgene plasmid # 61500 ; http://n2t.net/addgene:61500 ; RRID:Addgene_61500)[5]

pDO36 was cloned via Gibson Assembly[6] of four fragments. The vector backbone was amplified from E-cadherin-GFP with forward primer ATT TCC GGT TAA TAG AAT TCT AGA GGG CCC TAT TCT ATA GTG TCA CCT AAA TGC and reverse primer CCA CCG TAC ACG CCT ACC GCC CAT TTG CGT CAA TGG. From the same template plasmid the E-cadherin-Δcyto fragment was amplified with forward primer CCA TTG ACG CAA ATG GGC GGT AGG CGT GTA CGG TGG and reverse primer CTC CTC GCC CTT GCT CAC. The mCherry fragment was obtained from pDO13 (SNAPf-mCherry, unpublished data) using forward primer GTG AGC AAG GGC GAG GAG and revers primer CTT GTA CAG CTC GTC CAT GCC G. The $Halo_{stop}$ fragment was amplified from pDO35 (hE-cadherin-Δcyto-EGFP-Halo, unpublished data) with forward primer CGG CAT GGA CGA GCT GTA CAA G and reverse primer TTA GGT GAC ACT ATA GAA TAG GGC CCT CTA GAA TTC TAT TAA CCG GAA ATC TCC.

The plasmid pDO37 was created by inserting the cyto fragment into BsrGI and NotI linearized plasmid pDO28 (DHFR-EGFP, unpublished data). The cyto fragment was amplified from E-cadherin-GFP with forward primer GCA TGG ACG AGC TGT ACA AGG GAG GAG GAG GAA GTA AAG AGC CCT TAC TGC CCC CAG AGG and reverse primer GCT GAT TAT GAT CTA GAG TCG CGG CCT TAG TCG TCC TCG CCG CCT CCG TAC ATG TCA GC.

To clone pDO56 the plasmid coding for EcΔ-GFP-α(280-906) was linearized with BrsGI and SacII. DNA sequence for $Halo_{stop}$ was amplified from pDO34 (hE-cadherin-ΔβctnBS-EGFP-Halo, unpublished data) with forward primer CGG CAT GGA CGA GCT GTA CAA G and reverse primer GGA GAG GGG CGG ATC CTT AAG CAA TTG TCT AGA ATT CTA TTA ACC GGA AAT CTC C. The IRES fragment was amplified from pERB217 with forward primer GGA GAT TTC CGG TTA ATA GAA TTC TAG ACA ATT GCT TAA GGA TCC GCC CCT CTC C and reverse primer GGT GCG CTT CAT TTC GCA GTC TTT GTC CAT ATT ATC ATC GTG TTT TTC AAA GGA AAA CC. SNAP-mCherry was generated from pDO32 (SNAP-mCherry-β-catenin, unpublished data) using forward primer GGT TTT CCT TTG AAA AAC ACG ATG ATA ATA TGG ACA AAG ACT GCG AAA TGA AGC GCA CC and reverse primer GTC AAA GTT ATT GAG TGC ATA CCG CGG TGC AGA ATT CGA AGC TTG AGC TCG. The four fragments were ligated in a Gibson Assembly reaction.

pDO68 was assembled by inserting Halo-IRES and DHFR-EGFP-cytointo BmtI and XbaI linearized EcΔ-GFP-α(280-906). DNA fragment for Halo-IRES was amplified from pDO61 (Dual1-DHFR, unpublished data) with forward primer CCA GAG GAT GAC ACC CGG GCT AGC GAT AAC GAT GGA TCC GAA ATC GGT ACT GG and reverse primer CGC TAA CGC CGC AAT CAG ACT GAT CAT ATT ATC ATC GTG TTT TTC AAA GGA AAA CC. The DHFR-EGFP-cyto fragment was derived from pDO37 (DHFR-EGFP-cyto) using forward primer GGT TTT CCT TTG AAA AAC ACG ATG ATA ATA TGA TCA GTC TGA TTG CGG CGT TAG CG and reverse primer TGA CAC TAT AGA ATA GGG CCC TCT AGA GTC GCG GCC TTA GTC GTC CTC G.

3 NMR Spectra

^{1}H NMR (300 MHz, CD_3OD) of **Ha-BG (3)**

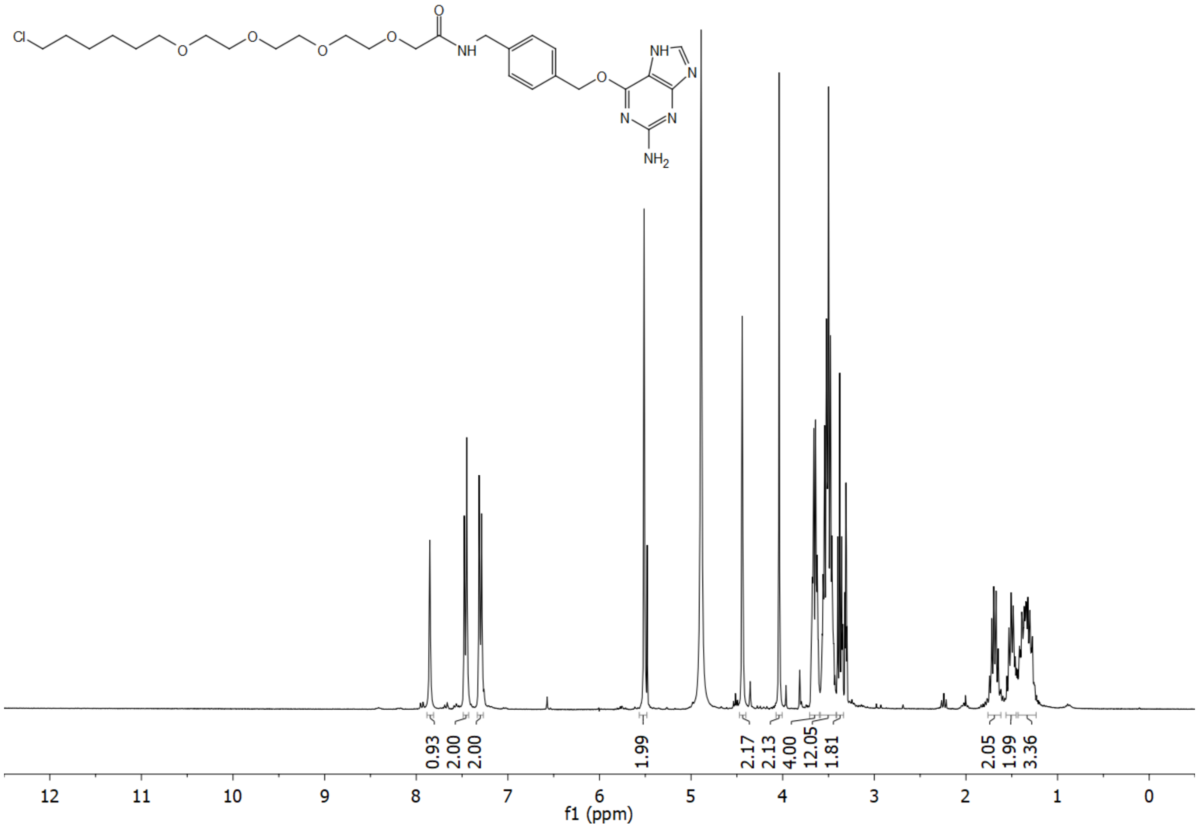

^{13}C NMR (75 MHz, CD_3OD) of **Ha-BG (3)**

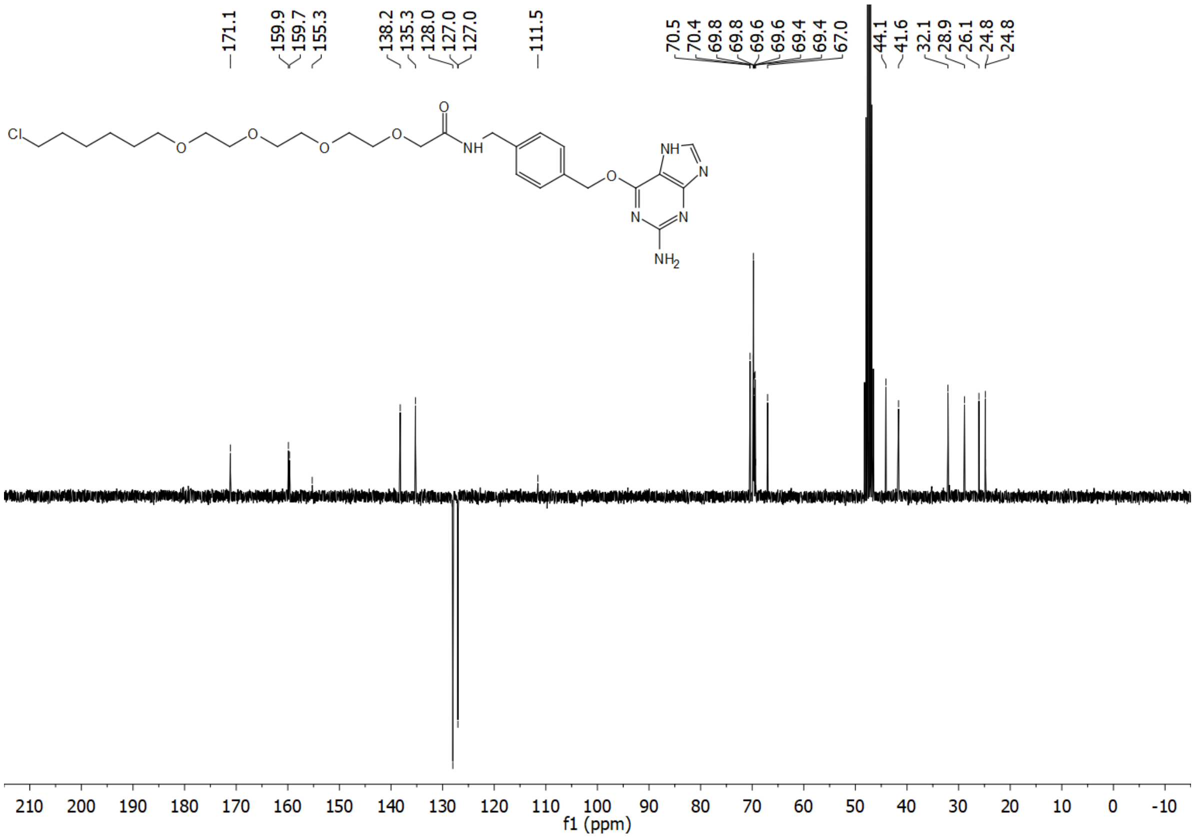

^{1}H NMR (300 MHz, CD_3OD) of **5**

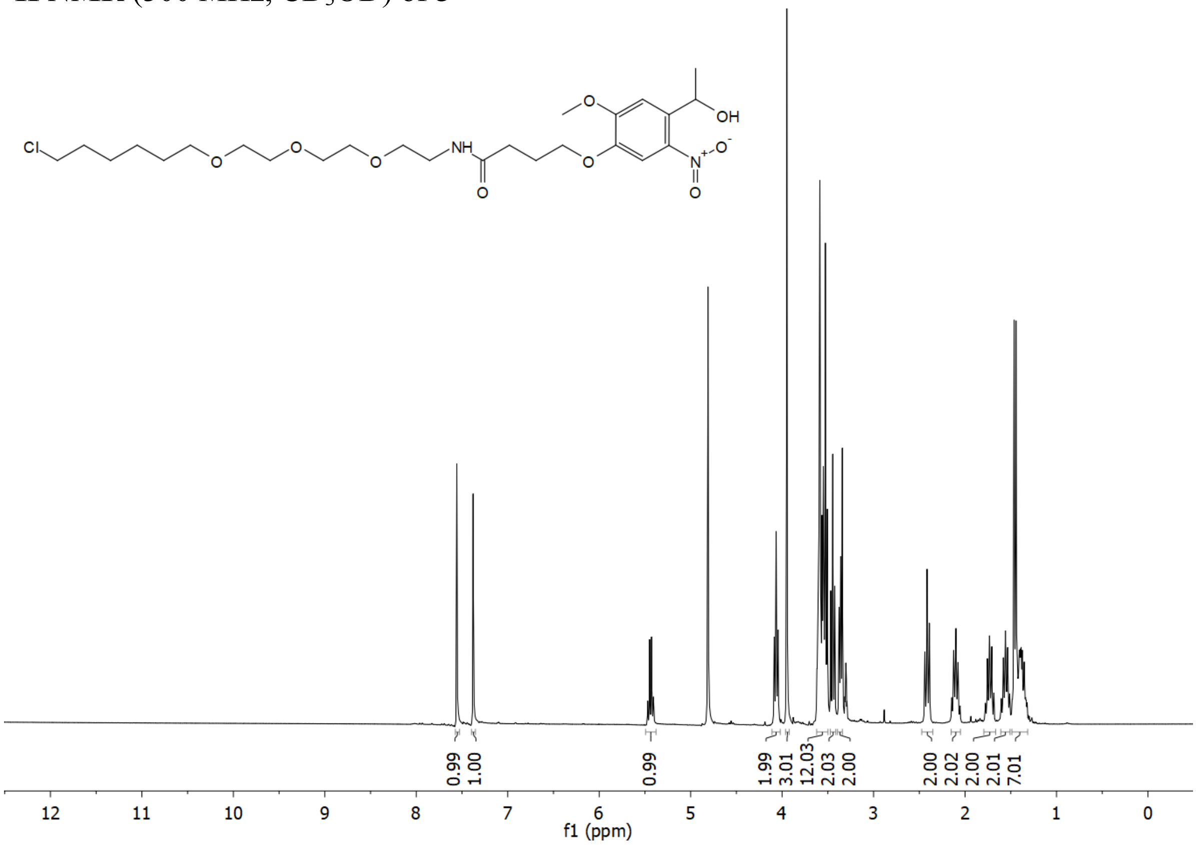

^{13}C NMR (75 MHz, CD_3OD) of **5**

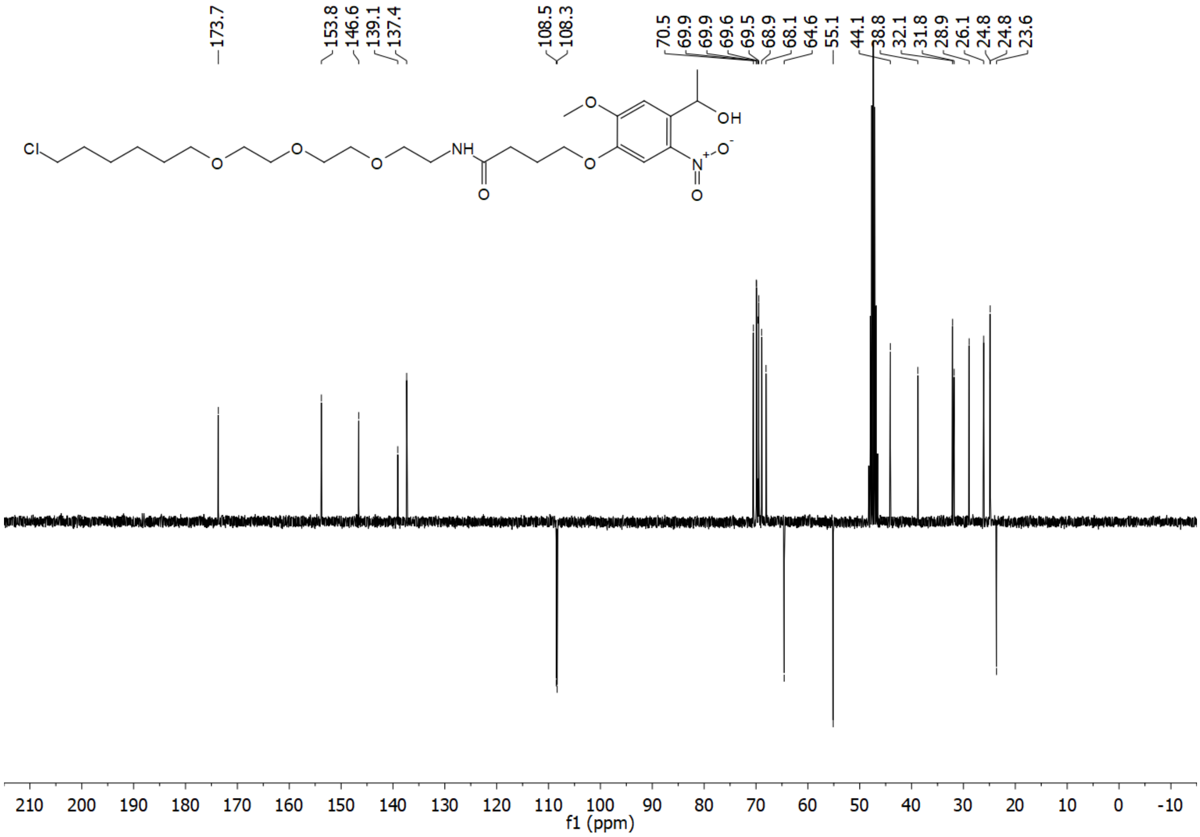

^{1}H NMR (300 MHz, CD_3OD) of **6**

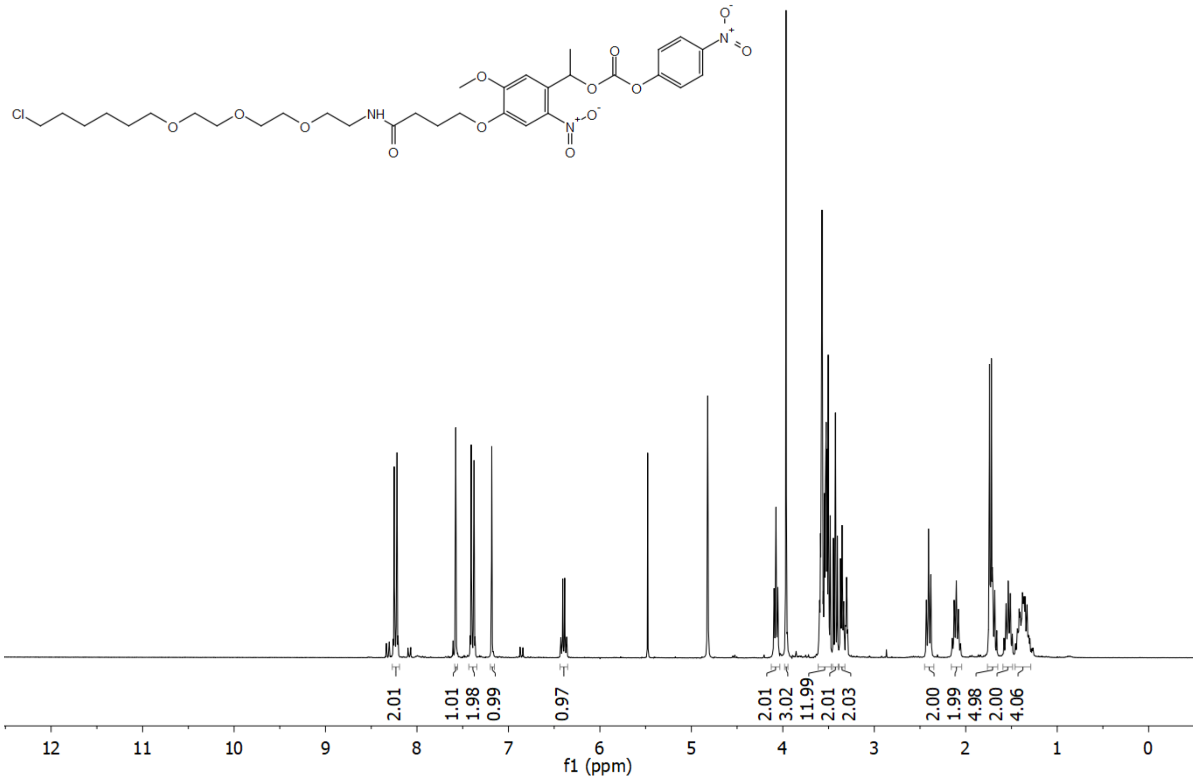

^{13}C NMR (75 MHz, CD_3OD) of **6**

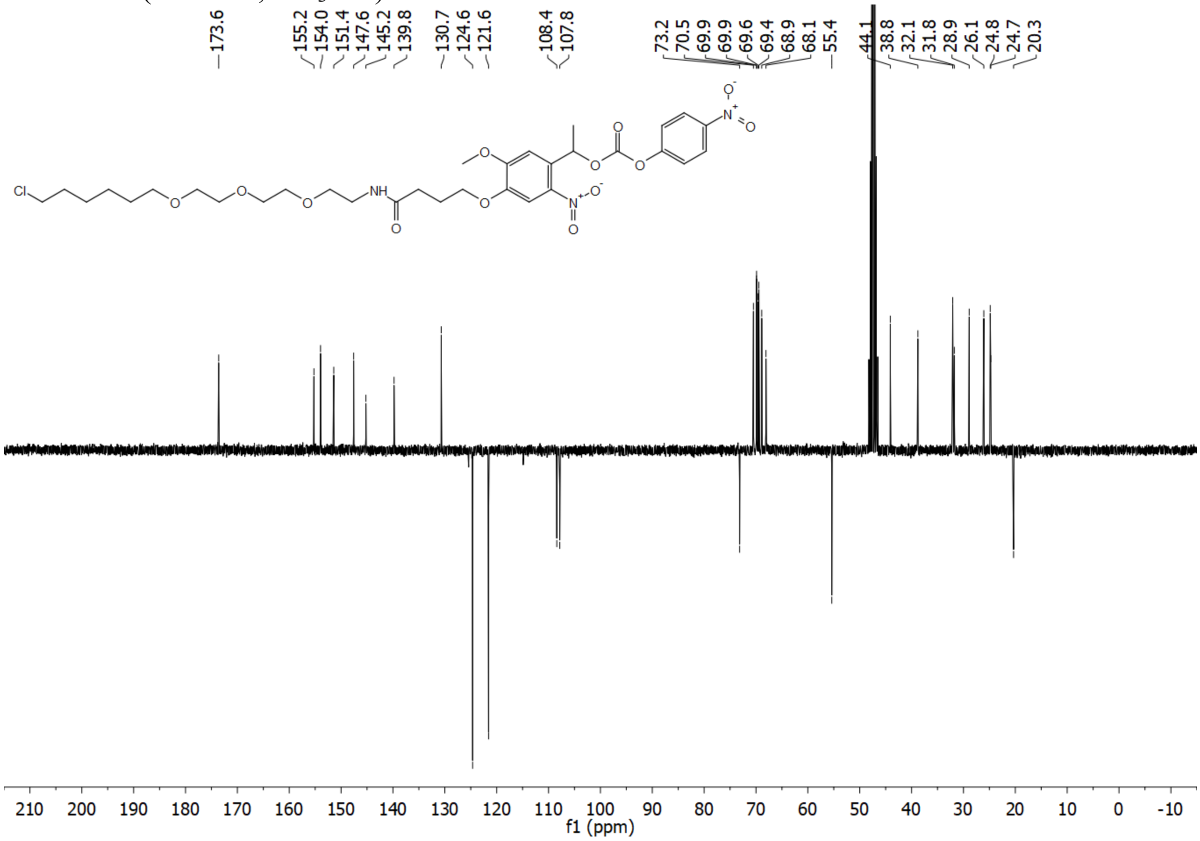

^{1}H NMR (500 MHz, CD_3OD) of **Ha-pl-BG (7)**

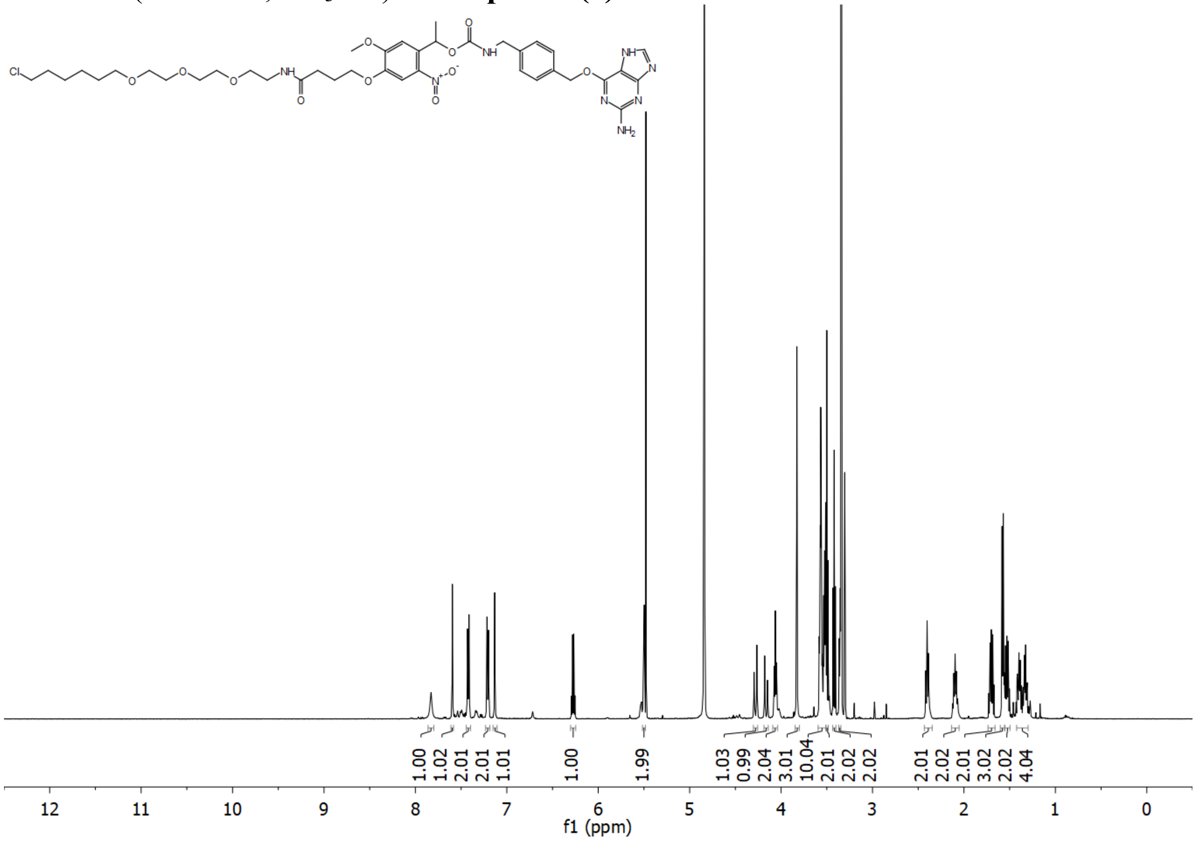

^{13}C NMR (125 MHz, CD_3OD) of **Ha-pl-BG (7)**

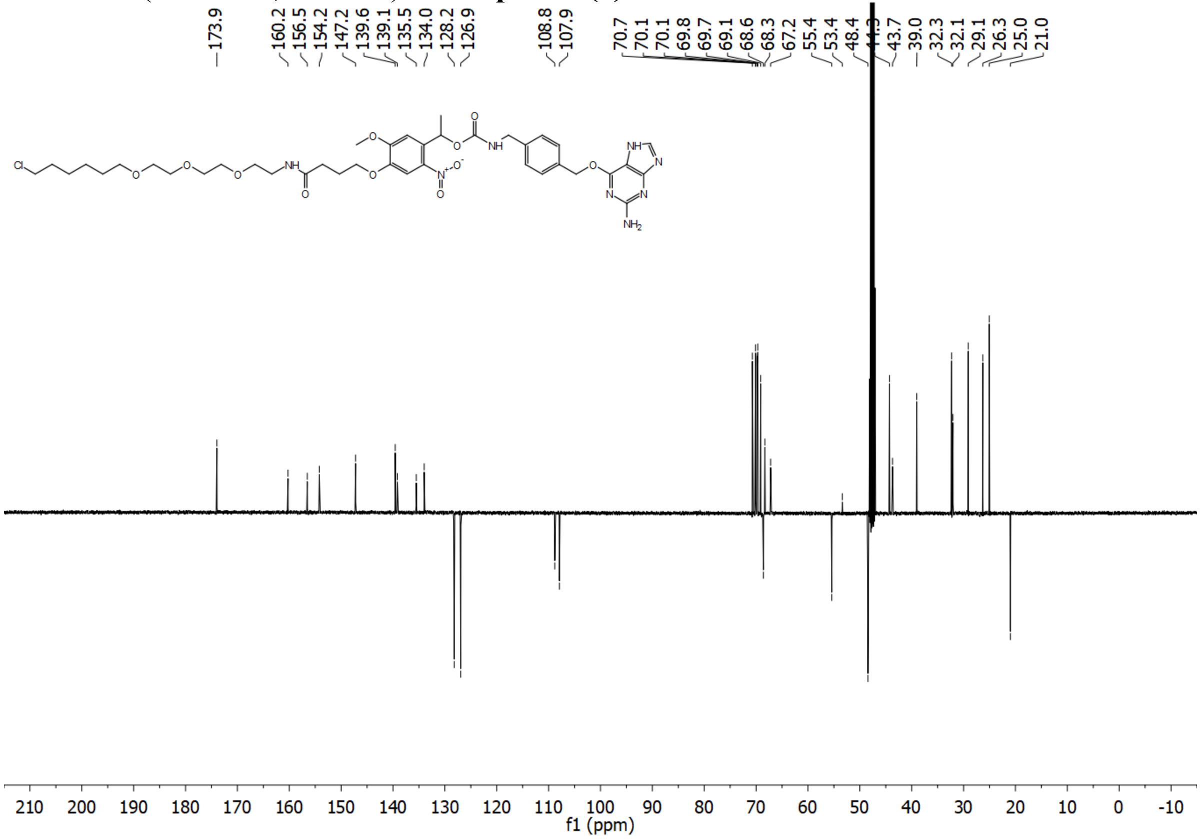

^{1}**H NMR** (500 MHz, CD_3OD) of **Ha- TMP (9)**

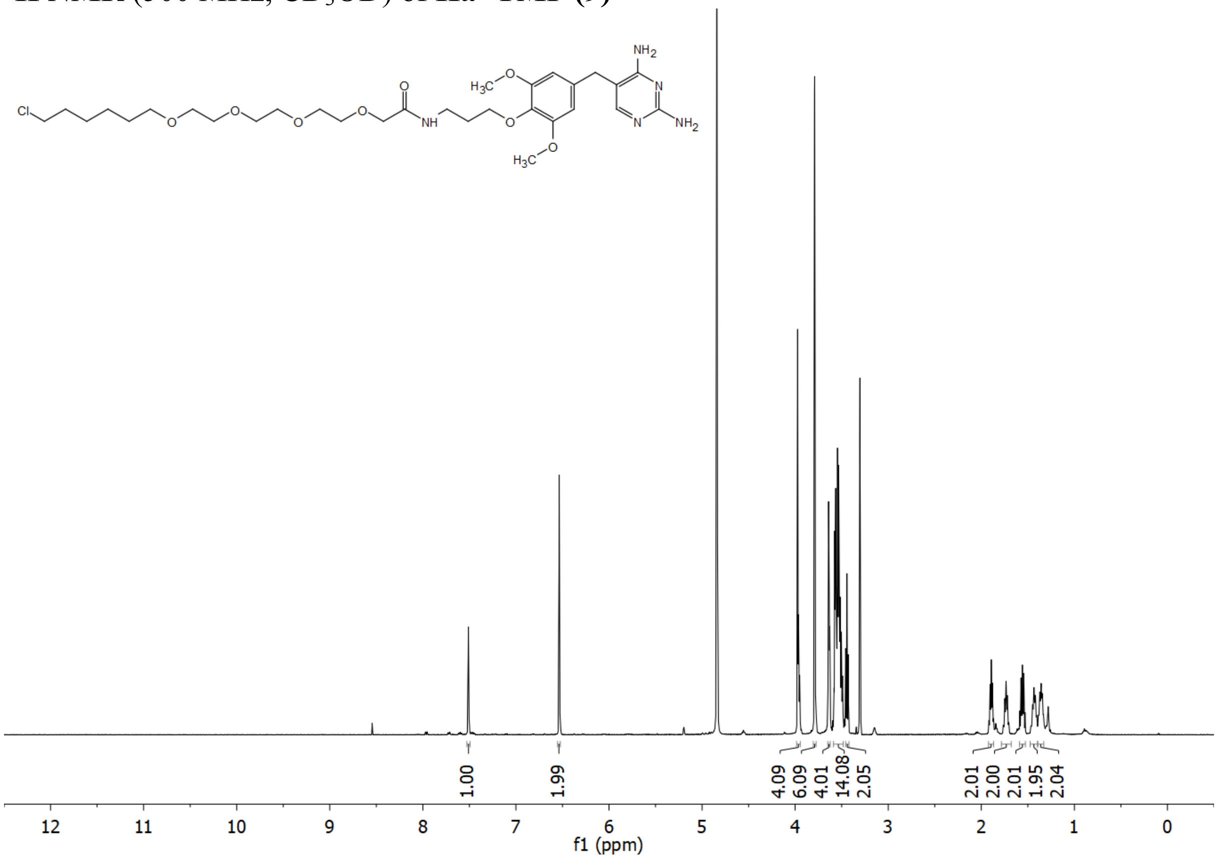

^{13}**C NMR** (126 MHz, CD_3OD) of **Ha- TMP (9)**

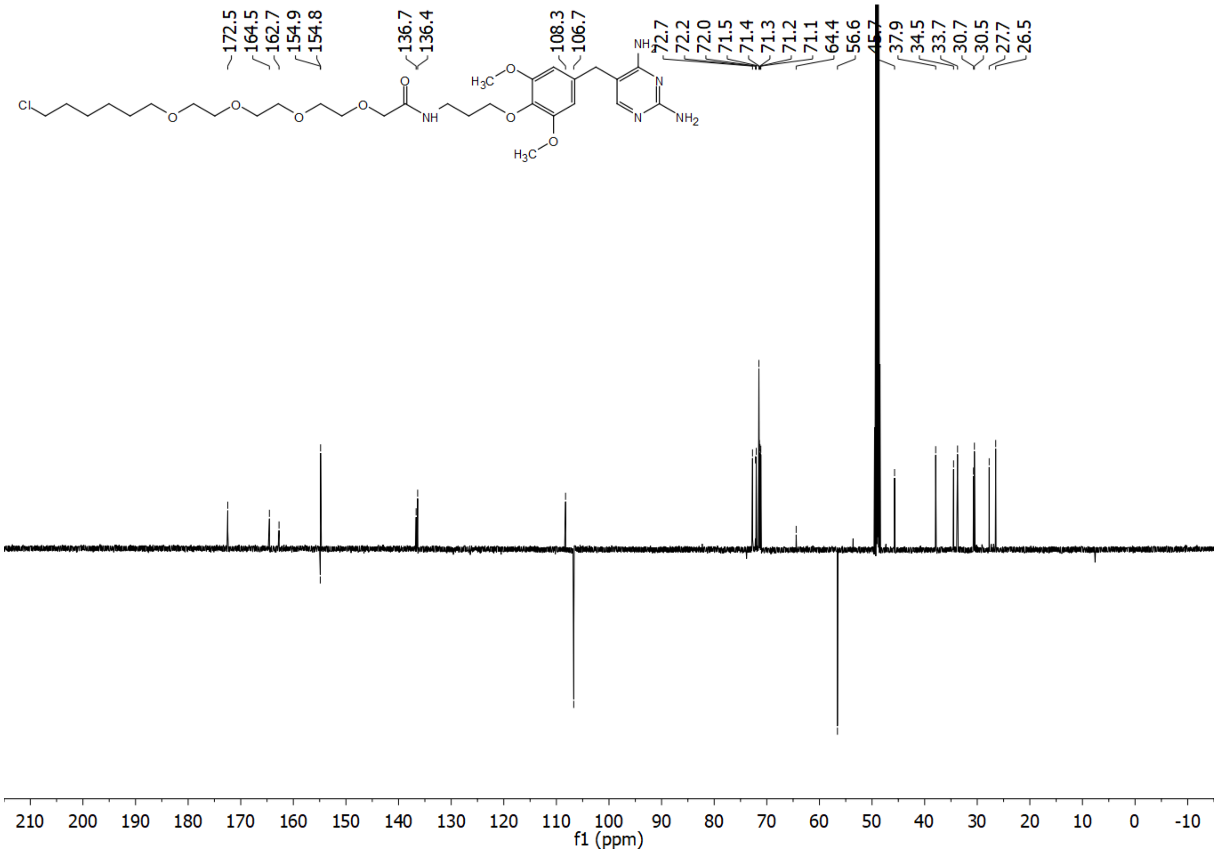

^{1}H NMR (300 MHz, CD_3OD) of **Ha-pl-TMP (10)**

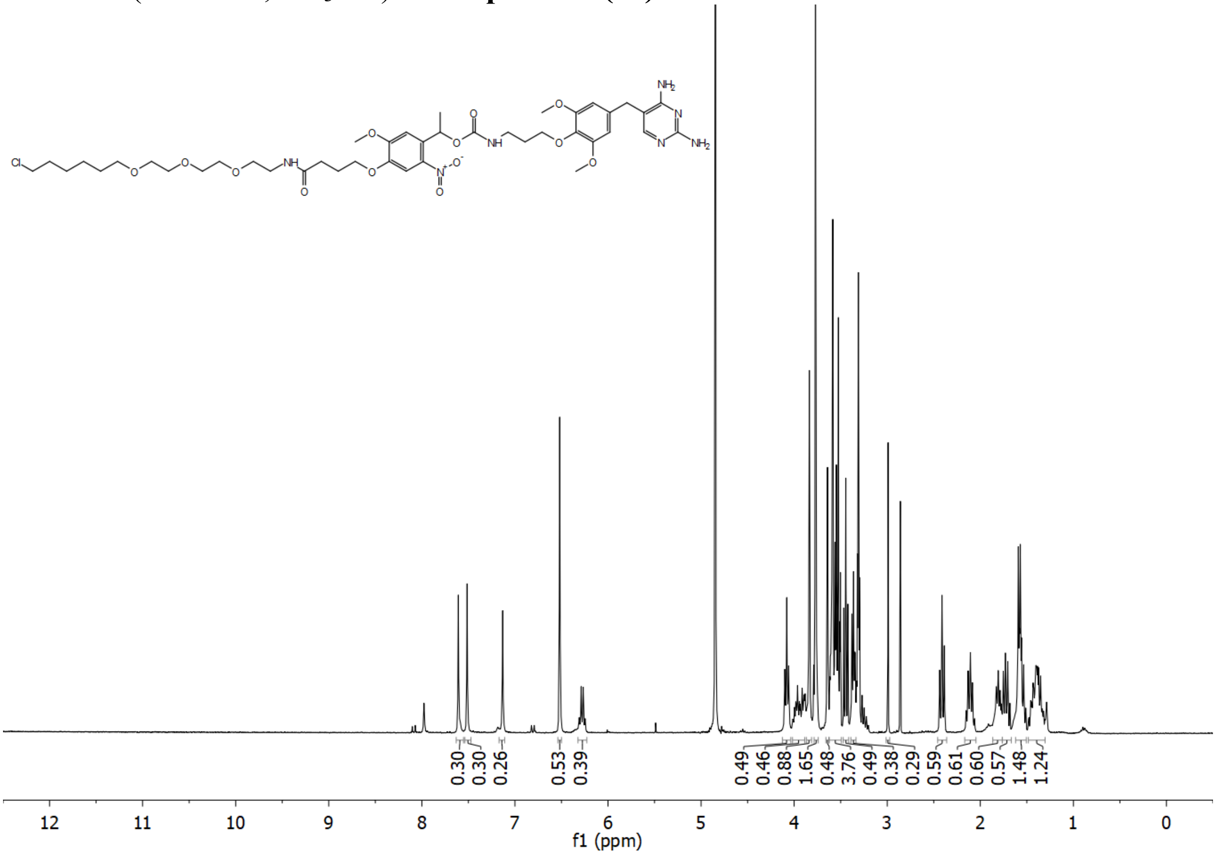

^{13}C NMR (75 MHz, CD_3OD) of **Ha-pl-TMP (10)**

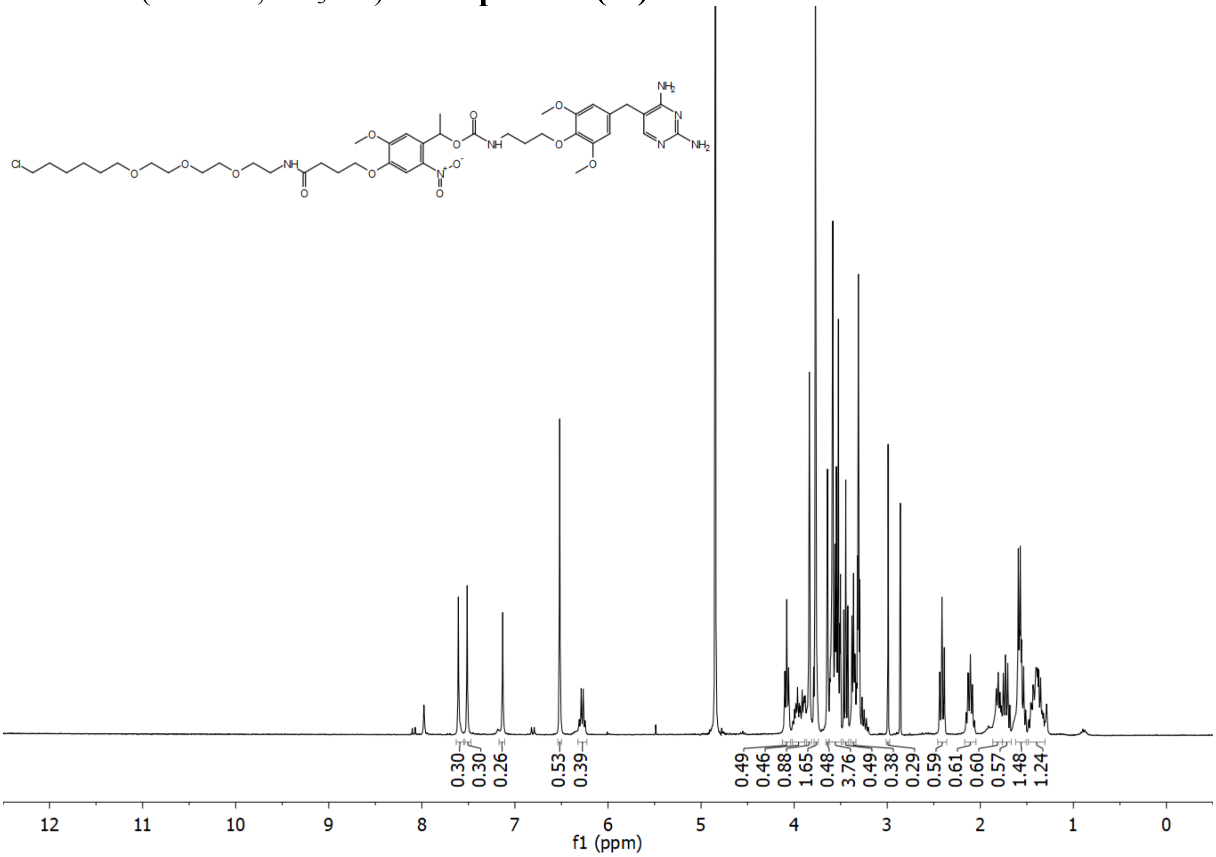

[1] G. R. Fulmer, A. J. M. Miller, N. H. Sherden, H. E. Gottlieb, A. Nudelman, B. M. Stoltz, J. E. Bercaw, K. I. Goldberg, *Organometallics* **2010**, *29*, 2176–2179.
[2] C. Jing, V. W. Cornish, *ACS Chem. Biol.* **2013**, *8*, 1704–1712.
[3] D. Erhart, M. Zimmermann, O. Jacques, M. B. Wittwer, B. Ernst, E. Constable, M. Zvelebil, F. Beaufils, M. P. Wymann, *Chem. Biol.* **2013**, *20*, 549–557.
[4] E. Fernandez-Megia, J. Correa, I. Rodríguez-Meizoso, R. Riguera, *Macromolecules* **2006**, *39*, 2113–2120.
[5] E. R. Ballister, C. Aonbangkhen, A. M. Mayo, M. A. Lampson, D. M. Chenoweth, *Nat Commun* **2014**, *5*, 5475.
[6] D. G. Gibson, L. Young, R.-Y. Chuang, J. C. Venter, C. A. Hutchison, H. O. Smith, *Nat Meth* **2009**, *6*, 343–345.
