# Supplementary material for: An optochemical tool for light-induced dissociation of adherens junctions to control mechanical coupling between cells
Source: Nat Commun. 2020 Jan 24;11:472. doi: 10.1038/s41467-020-14390-1 (PMC6981158; doi:10.1038/s41467-020-14390-1)
Supplement: Supplementary file 3 — Description of Additional Supplementary Files [file 41467_2020_14390_MOESM3_ESM.pdf]

Description of Additional Supplementary Files

Supplementary Movie 1: ***Light-induced dissociation of AJs with subcellular precision.***

Cells were preincubated with Ha-pl-BG to form AJs. SNAP-ΔN-α-catenin is shown in red, Ecadherin-Δcyto-Halo in green. Cells were imaged for 1 min before cutting the dimerizer to show the stability of the targeted AJ. Dynamics of cell-cell detachment can be followed after dissociation of the targeted AJs by scanning the white rectangular area in the black frame with the 405 nm laser. Notably, the non-targeted AJs are stable over the course of the time lapse recording. Images were recorded with an increased framerate for kymograph analysis (1 frame per 2 seconds for 00:40 seconds, then 1 frame per 10 seconds until 2:20 min) and reduced to 1 frame per 20 seconds thereafter to reduce photobleaching. For continuity reasons a framerate of 1 frame per 10 seconds was used for the video.

Supplementary Movie 2: ***Ha-pl-BG induced monolayer compaction and dissemination with spatial precision.***

Time lapse phase contrast imaging was started 1 h before addition of dimerizer to monitor the behavior of untreated cells (-01:00 h - -00:05 h). After Ha-pl-BG was added at 00:00 h, cells undergo a morphological change and integrate previously loosely attached cells in the monolayer. This leads to a compaction of the cell layer as indicated by reduced scattering of light. After 04:00 h maximum compaction is reached. At 04:10 h the area within the white rectangular was scanned with the 405 nm laser to cut the dimerizer and therefore dissociate the AJs. Cells within the targeted area immediately change back to a highly scattering appearance and push out the surplus of cells.

Supplementary Movie 3: ***Ha-pl-BG mediated reconstitution and light-induced dissociation of Adherens Junctions***.

Imaging was started directly after addition of Ha-pl-BG (-09:00 h) to follow the dimerizer-induced recruitment of the mCherry tagged SNAP-ΔN-α-catenin (red) to the GFP tagged E-cadherin-Δcyto-Halo (green). The E-cadherin-α-catenin complexes accumulate at cell-cell interfaces, indicating the formation of stable AJs. After 9 h an additional image was taken directly before and after illumination of the whole field of view with 350 nm light to show the immediate effect of photocleaving the dimerizer. Imaging was continued additional 4 h and during this period of time neither AJs assembly nor cell death were observed.

Supplementary Movie 4: ***Ha-BG mediated reconstituted of Adherens Junctions are stable after 350 nm light irradiation.***

The same conditions as described for Supplementary Video1 were applied, except that cells were incubated with the photostable dimerizer Ha-BG. In contrast to cells treated with Ha-pl-BG, AJs are stable even after illumination of the whole field of view with 350 nm light. Over a period of additional 4 h cell death did not take place and cells were rather undergoing cytokinesis, which led to dynamic rearrangement of AJs. AJs are reassembled as soon the cells start to spread after fission.

Supplementary Movie 5: ***Monolayer migration of Ha-pl-BG treated cells.***

After removing the confinement, dimerizer treated A431 α-catenin KO cells coexpressing E-cadherin-Δcyto-Halo and SNAP-ΔN-α-catenin migrate into the unoccupied glass surface area as a compact monolayer. Cell sheets flow like a fluid.

Supplementary Video 6. ***Monolayer migration of non-treated control cells.***

After removing the confinement, the same cells as described in Suppl. Vide 5 but not treated with dimerizer (negative control) migrate into the unoccupied glass surface area; the cell sheet moves in a less coordinated sand grain like flow compared to cells shown in Supplementary Video 6.

Supplementary Movie 7: ***Monolayer migration of Ha-pl-BG treated cells before and after light-induced dissociation of AJs*.**

After removing the confinement, dimerizer treated cells first migrate into the unoccupied glass surface area as a compact monolayer with fluid-like flow. After 2 h the central area marked with a white square in the black frame was scanned with the 405 nm laser to cut the dimerizer. Immediately after the targeted cells change their migratory behavior and become less coordinated as determined by PIV analysis.

Supplementary Movie 8: ***Traction force microscopy of a migrating monolayer of Ha-pl-BG treated cells before and after light-induced dissociation of AJs*.**

Transparent traction force profile overlaid on phase contrast images showing Ha-pl-BG treated cells migrating into the free gel surface after removing the confinement. Heat map color code was changed for better visibility of the phase contrast overlay. White arrows are traction force vectors indicating direction and amplitude of traction forces (corresponding with the heat map intensity scale). Traction forces progressively increase during the first hour and reach a steady state until the cells are irradiated with 405 nm laser to cut the dimerizer. Although the cell morphology changes immediately traction forces at the migration front are decreasing gradually.
